# Kinematic Responses to Changes in Walking Orientation and Gravitational Load in *Drosophila melanogaster*

**DOI:** 10.1371/journal.pone.0109204

**Published:** 2014-10-28

**Authors:** César S. Mendes, Soumya V. Rajendren, Imre Bartos, Szabolcs Márka, Richard S. Mann

**Affiliations:** 1 Department of Biochemistry and Molecular Biophysics, Columbia University, New York, New York, United States of America; 2 Neuroscience and Behavior at Barnard College, Columbia University, New York, New York, United States of America; 3 Department of Physics, Columbia University, New York, New York, United States of America; Alexander Fleming Biomedical Sciences Research Center, Greece

## Abstract

Walking behavior is context-dependent, resulting from the integration of internal and external influences by specialized motor and pre-motor centers. Neuronal programs must be sufficiently flexible to the locomotive challenges inherent in different environments. Although insect studies have contributed substantially to the identification of the components and rules that determine locomotion, we still lack an understanding of how multi-jointed walking insects respond to changes in walking orientation and direction and strength of the gravitational force. In order to answer these questions we measured with high temporal and spatial resolution the kinematic properties of untethered *Drosophila* during inverted and vertical walking. In addition, we also examined the kinematic responses to increases in gravitational load. We find that animals are capable of shifting their step, spatial and inter-leg parameters in order to cope with more challenging walking conditions. For example, flies walking in an inverted orientation decreased the duration of their swing phase leading to increased contact with the substrate and, as a result, greater stability. We also find that when flies carry additional weight, thereby increasing their gravitational load, some changes in step parameters vary over time, providing evidence for adaptation. However, above a threshold that is between 1 and 2 times their body weight flies display locomotion parameters that suggest they are no longer capable of walking in a coordinated manner. Finally, we find that functional chordotonal organs are required for flies to cope with additional weight, as animals deficient in these proprioceptors display increased sensitivity to load bearing as well as other locomotive defects.

## Introduction

Multi-jointed organisms move through their environment with a remarkable ability to adapt to internal physiological conditions, as well as to variations in terrain and other external forces such as wind or gravity. Such capabilities allow for locomotion that is fast, stable and energy efficient. Two features of animal nervous systems are critical for such remarkable adaptability. First, animals bear a large set of sensory structures specialized for sensing segment status and body position [1], [2]. For example, subsets of neurons in the second antennal segment of *Drosophila* are responsible for gravity perception. [3], [4]. Second, a complex neuronal architecture is able to receive and integrate internal and external sensory stimuli and produce an appropriately fine-tuned motor response. Some of these responses are reflex-based, comprising simple circuits such as the giant fiber system of insects that is responsible for visually induced escape behavior [5]–[8]. Other motor responses are instead computed by higher decision centers in the brain. For example, the protocerebral bridge, a central complex neuropil in the central brain, is required for the motor output used for crossing gaps in the terrain [9], [10].

Critical to locomotive coordination is the animal’s ability to compute both the direction and strength of the gravitational force in order to maintain posture and anchoring to the walking substrate [11]. This allows for maneuverability through a wide range of environments, both in air and on land. Arthropods and insects in particular experience radical variations in their environment, easily moving across challenging terrains such as ceilings and vertical structures. This resourcefulness is made possible by the use of a large number of legs and the presence of sophisticated surface attachment systems, which include claws and adhesive pads [12], [13]. Moreover, walking animals can sustain large variations in their body load, which becomes increasingly important under different physiological conditions such as obesity, or while carrying heavy objects [14]. For example, rhinoceros beetles can carry more than 30 times their body mass with notable metabolic economy [15]. During the step cycle, each leg supports load variation, with maximum support occurring halfway through the stance phase [16], [17].

In order to deal with variations in body load and orientation, vertebrates and invertebrates resolve gravity information through direct and indirect approaches. Mammals and insects can detect linear accelerations using the inner ear and the antennal Johnston’s organ, respectively [18], [19]. Particular structures in the mechanosensory system can measure joint angles and strains, providing additional information about the direction and strength of the gravitational force. In mammals these structures include muscle spindles and Golgi tendon organs [20]. In insects, these include hair plates, chordotonal organs and campanifom sensilla [1] – sensory organs specialized to meet a different set of mechanical challenges from those faced by mammals [21]. A large body of work has focused on the role of campanifom sensilla in transducing leg load during standing and walking conditions [22]–[25]. These are dome-like structures present in legs that can be isolated or grouped. Their ciliated dendrites are mechanically coupled with the cuticle, allowing for detection of mechanical stress in the exoskeleton [1]. Although there is some variability among insect species in the position of the campaniform sensilla, they are generally placed and oriented strategically close to leg joints, making them particularly sensitive to force orientation or phase of the step cycle [26]. For example, in the cockroach tibia, proximal campaniform sensilla respond to force increases during load bearing, whereas distal receptors fire to decreasing force as legs unload [24]. These afferents project into the ventral nerve cord where they can synapse with motor neurons as well as spiking and nonspiking interneurons [27], [28]. Direct synapse onto motor neurons can work as a negative feedback system in order to lessen the mechanical stress that caused the initial sensilla to fire [29]. This can be done by exciting and simultaneously inhibiting a set of antagonistic leg muscles [30]. Moreover, campaniform sensilla can also influence motor activity by targeting Central Pattern Generators (CPGs) [30], [31].

Motor adaptability is reflected in the timing and intensity of muscle contractions in response to terrain conditions, body load and orientation relative to gravity [32]. Although experiments have been designed to challenge animals during walking, very few studies have focused on the kinematic responses to changes in walking conditions. One reason for this is the difficulty in extracting large kinematic data sets from freely moving animals. Instead, a large body of work has focused on kinetics (i.e. force distribution, for example [33], [34]) or electrophysiological responses to body load (for example [23], [25], [35] reviewed in [32]). Due to its small size, the fruit fly remains technically challenging for physiology and force distribution (kinetics) studies compared to the stick insect, cockroach or locust. Nevertheless, an increasingly sophisticated genetic toolkit makes this a valuable model system to identify and manipulate circuits involved in motor control with increased cellular resolution [36]. In addition, the fruit fly displays an extremely reliable, stereotyped and stable locomotor behavior that is dependent on walking speed [37]–[39].

Machine vision techniques that track untethered animals can quantify a large number of locomotor parameters and provide a detailed behavioral readout of wild type and genetically manipulated animals [39]–[41]. Here, we took advantage of one of these methods, which combines a high-speed optical touch sensor with a tracking software package that allows the extraction of a large number of kinematic parameters with high spatial and temporal resolution [39]. Using this method, we asked what kinematic parameters change as a result of different walking orientations and increased gravitational load. We find that although many of the rules governing walking behavior are unaffected, changes in orientation and load alter the walking program to promote gait stability at the expense of walking speed. Moreover, we identify a threshold for carrying additional weight beyond which flies are unable to manage. Importantly, we also find that some responses to increased weight vary over time, suggesting that distinct neural control mechanisms mediate short and long term responses.

## Results

### Kinematic responses to walking orientation

**Figure 1:**
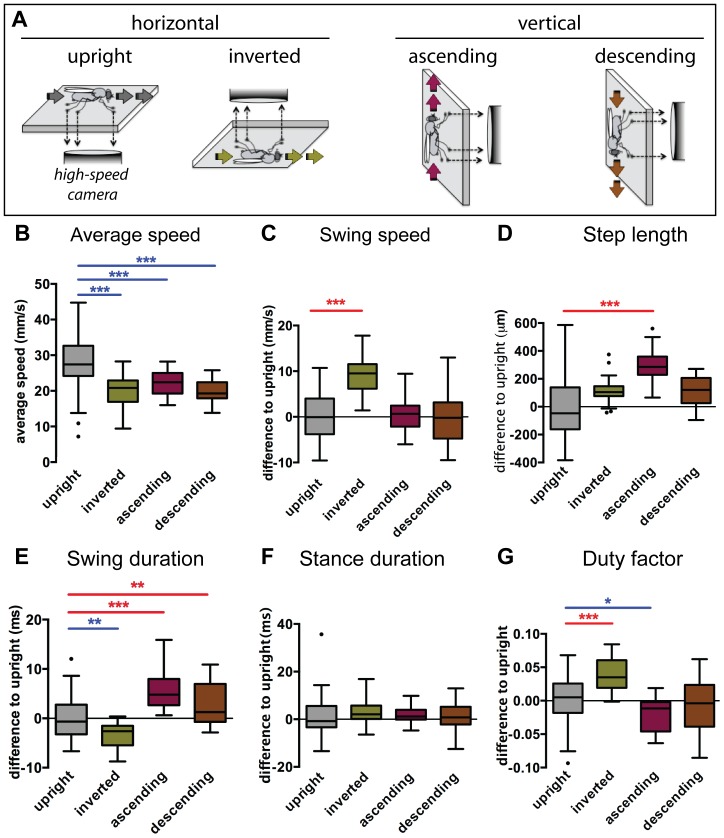
Imaging setup and step parameters. (A). Schematic of the recording setup. The fTIR apparatus was oriented in order to allow the flies to walk freely inside a walking chamber either horizontally (upright or inverted) or vertically (ascending or descending). A high-speed camera recorded body and tarsal contacts on the optical glass, see [39] for details. Fly schematic adapted from [73]. (B–G) Boxplots represent the median as the middle line, with the lower and upper edges of the boxes representing the 25% and 75% quartiles, respectively; the whiskers represent the range of the full data set, excluding outliers. Circles indicate outliers. Grey represents upright controls (n = 71, from [39]), green for inverted (n = 28), purple for ascending (n = 28) and brown for descending (n = 23). For C–G data was residual normalized and expressed as the difference to the upright control. Statistical analysis with one-way ANOVA followed by Tukey’s *post hoc* test (for normal distributions in panels B, C and E) or Dunn’s *post hoc* test (for non-normal distribution in panels D, F and G), ^*^ *P*<0.05; ^**^ *P*<0.01; ^***^ *P*<0.001. Statistically significant increases or decreases are indicated in red and blue, respectively. (B) Non-upright animals display significantly reduced walking speed. (C) Swing speed was significantly increased in inverted walking animals. (D) All non-upright animals display increased step length. (E) Swing duration is increased only in ascending and descending animals while inverted animals show a small decrease. (F) Stance duration remains unchanged for all conditions. (G) Duty factor strongly increases in inverted walking animals while it is minimally reduced in ascending animals.

In order to quantify locomotion in different orientations we used the FlyWalker system [39], reorienting the detection apparatus to allow footprint detection while animals walked upside down (inverted) or vertically (ascending or descending; Figure 1A). Animals were allowed to walk freely and the walking kinematics were quantified as previously described [39]. Briefly, this method is based on the reflection of light within an optical glass through an optical effect termed *Total Internal Reflection* (TIR). Leg contacts disrupt this effect causing *frustrated TIR* (fTIR), which generates scattered light that can be detected by a high-speed camera. Using custom-made software specifically developed for this assay, we were able to track, quantify and compare the walking pattern between different conditions. All kinematic definitions are described in Table S1.

#### Step related parameters

Animals walking in non-upright conditions displayed significantly slower walking speeds and narrower range of values for this parameter compared to upright controls, suggesting tighter motor control (Figure 1B). We next asked if step parameters were solely dependent on the walking speed as previously described [37]–[39], or if in addition orientation might influence walking as previously observed in the locust or cockroach [42], [43]. We found that not only did step parameters change under non-upright conditions but that the degree of modulation was dependent on the walking orientation. Swing speeds were significantly increased in animals walking in the inverted orientation, while in vertically walking animals (ascending or descending) this parameter remained unchanged (Figure 1C and S1A). Interestingly, in both ascending and descending conditions, animals displayed a proportional increase in step length and swing duration (Figure 1D, E and S1B, C), explaining the unchanged swing speed. In contrast, inverted animals not only displayed a slight increase in step length but also had shorter swing phases, thus explaining the increased swing speed (Figure 1D, E and S1B, C). This result is similar to that observed in cockroaches, which display shorter swing phases while walking inverted and supported by a fine mesh [43]. In contrast to swing parameters, stance phase parameters displayed the same speed-dependent response in all tested conditions (Figure 1F and S1D), suggesting that adaptability to walking orientation targets mostly the swing phase of the step cycle. Because of a shorter swing phase (Figure 1E and S1C), the duty factor, which measures the fraction of the step cycle dedicated to the stance phase, was significantly increased in inverted animals, indicating, on average, an increase in contact with the substrate. In contrast, vertically walking animals had minimal or no change in this parameter (Figure 1G and S1F). Overall, our results indicate that flies have the capacity to adapt their step parameters in accordance with their walking orientation by shifting their stride length and protraction duration.

#### Spatial parameters

**Figure 2:**
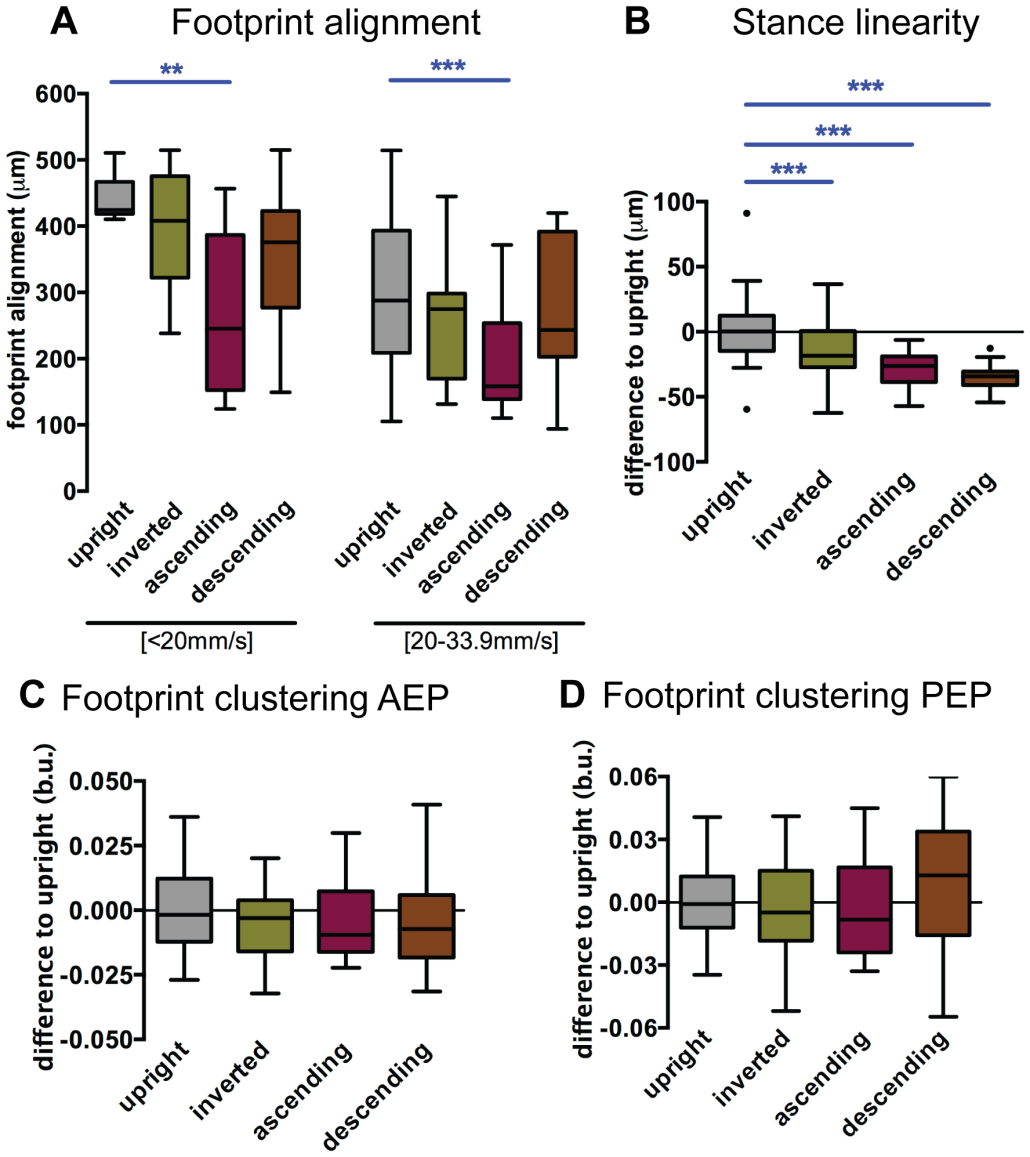
Spatial parameters. Non-upright walking animals display more aligned footprints and less jitter during stance phases, while clustering remains unchanged. (A–D) Boxplots represent the median as the middle line, with the lower and upper edges of the boxes representing the 25% and 75% quartiles, respectively; the whiskers represent the range of the full data set, excluding outliers. Circles indicate outliers. Statistical analysis with one-way ANOVA followed by Tukey’s *post hoc* test, ^*^ *P*<0.05; ^**^ *P*<0.01; ^***^ *P*<0.001. Statistically significant decreases are indicated in blue. In B, C and D data was residual normalized and expressed as the difference to the upright control. (A) Ascending animals show more aligned footprints. Data were grouped into slow (<20 mm/s) and medium (between 20 and 34 mm/s) speeds. (B). Non-upright animals display lower values for stance linearity indicating lower jitter during stance phases. (C–D) Footprint clustering remains unchained for Anterior Extreme Position (AEP) and Posterior Extreme Position (AEP). (b.u., body units).

FlyWalker also quantifies the footprint positions relative to the center of the body. The footprint alignment parameter, also called follow-the-leader, measures the propensity for all three ipsilateral legs to be placed in a similar position relative to the walking vector [39], [44], [45]. Since we previously observed that this parameter distributes in a non-linear fashion for upright animals [39], we analyzed the data for two speed groups, slow (<20 mm/s) and medium speeds (20–34 mm/s) (in our experiments, non-upright flies did not walk faster than 34 mm/s; Figure 2A and S2A). Animals ascending a vertical plane showed an increased tendency to align their footprints, perhaps as an effort to ensure secure anchoring. In inverted or descending animals, no significant difference was detected, possibly as a consequence of other gait features that prevent a change in footprint alignment.

We next measured the stance linearity index, which quantifies the steadiness of tarsal contacts relative to the body center while the fly moves forward [39]. Interestingly, animals walking in non-upright conditions exhibited smaller stance linearity values (Figures 2B and S2B), indicating steadier stance phases in the face of more challenging conditions. In addition, no significant changes were observed in footprint clustering, both during footfall (anterior extreme position; AEP) or swing onset (posterior extreme position; PEP) [46] (Figures 2C,D and S3). Moreover, descending animals showed a consistent posterior displacement of AEP and PEP positioning, possibly as a consequence of gravitational pull on the fly’s body (Figure S4). Together, our results indicate that the fruit fly engages in a more controlled stance phase (e.g. lower stance linearity values) during non-upright conditions, although footprint placement precision (AEP and PEP clustering) remains unaffected. These results differ from the stick insect that displays strikingly different footprint positions depending on the walking orientation [46].

#### Coordination parameters

**Figure 3:**
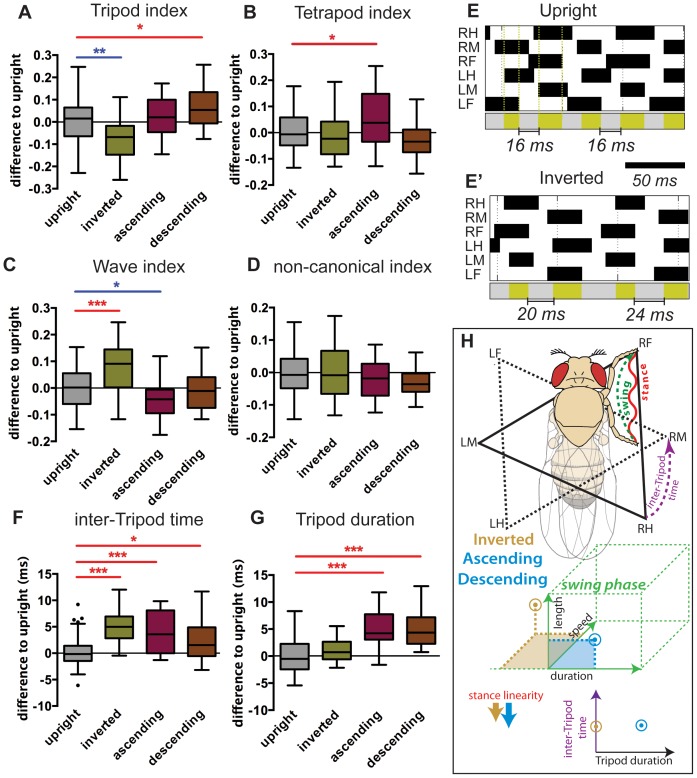
Interleg coordination parameters and summary of kinematic effects in non-upright walking animals. (A–D, F–G) Boxplots represent the median as the middle line, with the lower and upper edges of the boxes representing the 25% and 75% quartiles, respectively; the whiskers represent the range of the full data set, excluding outliers. Circles indicate outliers. Data was residual normalized and expressed as the difference to the upright control. Statistical analysis with one-way ANOVA followed by Tukey’s *post hoc* test, ^*^ *P*<0.05; ^**^ *P*<0.01; ^***^ *P*<0.001. Statistically significant increases or decreases are indicated in red and blue, respectively. (A) Inverted walking animals display a significant decrease in tripod configurations while descending animals showed an increase. (B) Only ascending animals displayed a slight increase in tetrapod configurations. (C) Inverted walking animals display a significant increase in the use of wave conformations while ascending animals showed a slight decrease. (D) No variation was observed in the number of non-canonical combinations for all experimental groups. (E) Step patterns and tripod/transition phases. (E) and (E’) display two representative videos of animals walking upright and inverted at similar speeds, respectively. In the upper section, for each leg, swing phases are represented in black (from top to bottom: right hind (RH); right middle (RM); right front (RF); left hind (LH); left middle (LM); left front (LF)). Vertical dashed green lines represent the boundaries of a tripod stance phase. Lower section represents the periods associated with tripod and transition (or *inter-Tripod time*) phases depicted in green and grey, respectively. (F) Non-upright walking animals display a significant increase in inter-Tripod time. (G) Animals walking in a vertical plane display an increase in the average duration of each tripod stance phase. (H) Summary of kinematic affects under non-upright conditions. For simplicity, only the step cycle of the right foreleg is represented. Dashed green line represents the swing phase from PEP to AEP. Waved red line represents stance traces. Solid triangle represents the tripod conformation formed by RF, LM and RH. Dashed triangle represents the immediately subsequent tripod conformation. Purple dashed line represent the inter-Tripod time between the two tripod conformations. Brown and blue arrows represent the qualitative variations compared to upright walking animals observed for inverted and ascending/descending, respectively.

Interleg coordination is critical for sustaining speed, stability and energy efficiency during walking [38], [39], [47]–[49]. *Drosophila* use tripod coordination as the preferred gait, particularly at higher speeds [37]–[39]. Alternatively, at very low speeds, tetrapod and wave (or metachronal) patterns can also be observed where four or five legs are in stance position, respectively [38], [39], [50]. We reasoned that flies might shift their gait under different walking orientations. To test this we calculated the fraction of frames in which leg contact was associated with a particular gait, also known as gait indexes [39] (Figure 3A–D). We found that flies walking in an inverted orientation significantly reduced their usage of the tripod gait while increasing the wave configuration (Figure 3A, C). Further, by visually inspecting the gait patterns of inverted walking animals we noticed an increased occurrence of the wave gait, where only a single leg swings at a given time [50], particularly at slow speeds (data not shown). Ascending flies also increased the use of tetrapod configurations while reducing the amount of the wave configuration (Figure 3B, C). These differences were particularly striking at slower speeds, when walking is more susceptible to neuronal control by higher control centers [51] (Figure S5). On the other hand, no changes were observed in the frequency of non-canonical configurations (Figure 3D).

One possibility to explain a reduced use of tripod configuration in inverted flies would be that animals under these conditions increase the amount of time separating two consecutive tripod stances. In order to test this possibility we analyzed two new parameters termed *inter-tripod time* that measures the time transitioning between two complementary tripod stances; and *Tripod duration*, which measures the average duration of each tripod stance (see methods section for details) (Figure 3E–G). Interestingly, faster animals display reduced inter-tripod transition times, with values of ∼10 ms for fast animals compared to ∼20 ms for slow animals (Figure S6A). In general, animals walking under non-upright conditions had larger *inter-tripod transition* values (Figure 3E, F and S6A). In addition, *Tripod duration* values were on average ∼4 ms longer for ascending or descending flies compared to upright flies (Figure 3G and Figure S6B), consistent with an increase in step length under these conditions (Figure 1D). Phase plots to examine the coordination between left-right or adjacent ipsilateral leg pairs were generated but did not reveal anything beyond these conclusions (data not shown).

Overall, our data indicate that *Drosophila* can readily modify its walking behavior according to orientation in order to sustain stability and contact to the substrate (major kinematic shifts are summarized in Figure 3H). These changes target the step cycle leading to faster leg swings or longer strides while walking upside down or vertically, respectively. In addition, interleg coordination properties are also adjusted under different walking orientation in order to maximize contact with the substrate.

### Kinematic responses to increased body load

**Figure 4:**
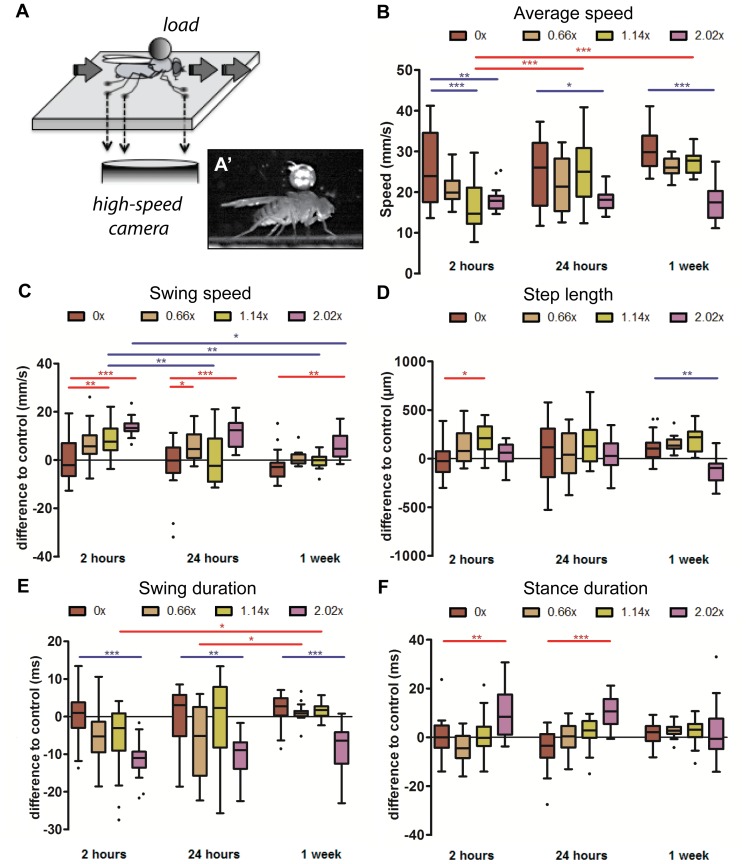
Imaging setup and step parameters. (A) Recording setup. Flies were allowed to walk freely inside a walking chamber on an optical glass with a metal ball bearing attached to the notum. A high-speed camera recorded body and tarsal contacts on the optical glass. (A’) Representative image of a fly carrying a metal ball bearing. (B–F) Box plots represent the median as the middle line, with the lower and upper edges of the boxes representing the 25% and 75% quartiles, respectively; the whiskers represent the range of the full data set, excluding outliers. Circles indicate outliers. Statistical significance was determined using 2-way-ANOVA with post-hoc t-tests, where ^*^ *p*<0.05; ^**^ *p*<0.01; ^***^ *p*<0.001. Statistically significant increases or decreases are indicated in red and blue, respectively. (B) Average Speed. Flies bearing 0.66× and 1.14× show a recovery in average speed by one week, while flies bearing 2.02× do not. (C) Swing speed. The immediate effect of weight is an increase in swing speed, although it decreased to that of controls for flies bearing 0.66× and 1.14× by 1 week. (D) Step length. Step length shows weight-dependence only, where intermediate weights increases step length at 2 hours and the heaviest weight decreases step length at 1 week. (E) Swing duration. Swing duration is significantly decreased for flies bearing 2.02×. (F) Stance duration. Stance duration is increased only for the heaviest weight.

In order to study the kinematic effects of body load on fruit flies we glued 1/32″ spherical ball bearings with different densities to the notum of wild type female flies (Figure 4A and Video S1). Weights corresponded to 0.66, 1.14 and 2.02 times an average body mass (female flies weighed 1.14±0.05 mg, n = 39). Animals with only glue on their notums, used to adhere the ball bearings, were used as controls. In addition, to study the long-term effects of load bearing we analyzed walking by these animals 2 hours, 24 hours and one week after attachment.

#### Step related parameters

We observed that weights affected locomotion and kinematic parameters, particularly above a weight threshold. Furthermore, some parameters changed over time. For example, flies bearing weights showed a decrease in speed 2 hours after the weight was added, not walking faster than 30 mm/s (Figure 4B). Moreover, flies bearing 0.66× and 1.14× weights slowed initially but eventually returned to control speeds. Flies bearing the heaviest 2.02× weights, however, did not recover, suggesting the existence of a threshold for weight tolerance that exists between 1.14× and 2.02× of body mass.

Some step parameters were also affected by weight bearing over time. In general, swing speeds were increased (Figure 4C). However, over time, this increase was only maintained for the heaviest loads; flies carrying lighter and intermediate weights returned to control values at later time points. Step length remained relatively constant (Figure 4D), except for flies bearing the heaviest weights for which it was strongly reduced. A similar response was observed for swing duration where lighter and intermediate weights remained unaffected while animals carrying heavier loads displayed a strong reduction in swing phase (Figure 4E). Animals under these conditions displayed on average a protraction that was approximately 10 ms shorter compared to control animals (Figure 4E), thus explaining the observed increase in swing speed (Figure 4C). Consistently, animals carrying the heaviest loads showed an increased duty factor at all time points (Figure S7B), possibly to achieve greater stability. Interestingly, the persistent decrease in swing duration for 2.02× weight bearing flies was coupled with an increase in stance duration (Figure 4F), allowing these flies to maintain a relatively constant step period (Figure S7B).

Together, these data show that fruit flies adapt their step parameters in response to increased weight. For animals bearing weights of 1.14× or below, responses are typically transient; flies can eventually adapt to carrying these loads and return to wild type step parameters. In contrast, flies bearing a weight of 2.02× display stronger and long-term effects, generally target swing duration by increasing swing speed.

#### Spatial parameters

**Figure 5:**
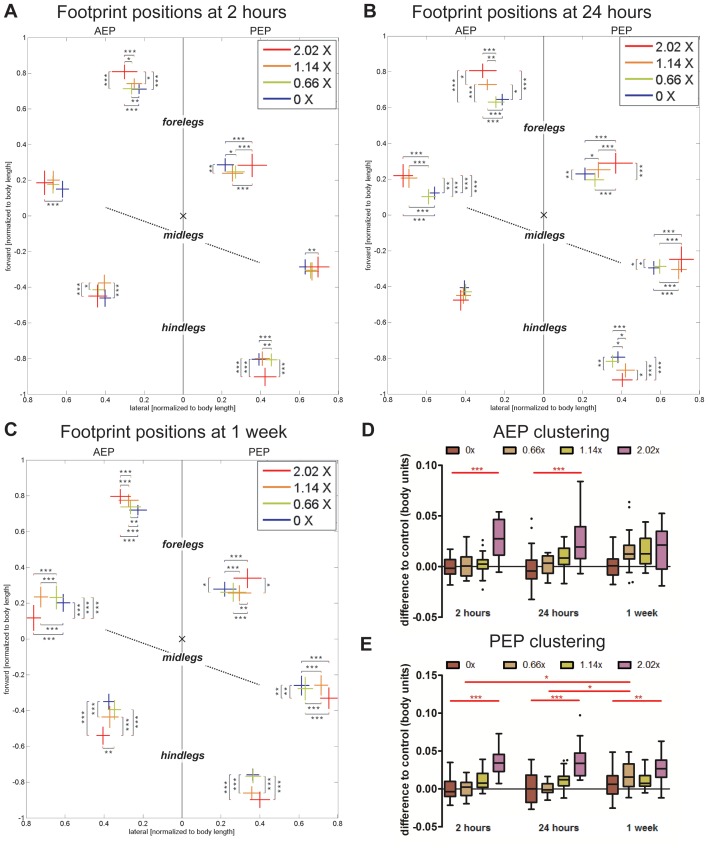
Spatial parameters. (A–C) Footprint positions relative to the body center. AEP and PEP values for each leg are represented on the left and right sections of the plot, respectively. Values are normalized for body size. Line size denotes standard deviations, while intersection indicates mean value. Statistical significance was determined using 2-way-ANOVA with Tukey’s post-hoc tests and post-hoc t-tests, where ^*^ *p*<0.05; ^**^ *p*<0.01; ^***^ *p*<0.001. Statistically significant increases are indicated in red. (D–E) AEP and PEP Clustering. Box plots represent the median as the middle line, with the lower and upper edges of the boxes representing the 25% and 75% quartiles, respectively; the whiskers represent the range of the full data set, excluding outliers. Circles indicate outliers. Values show a significant footprint dispersal in 2.02× bearing flies compared to controls.

Spatial parameters also changed in response to additional weight (Figure 5). For these measurements we plotted the AEPs (beginning of stance phase) and PEPs (beginning of swing phase) for each leg in relation to the center of the body (Figure 5A–C). We observed a significant displacement of these parameters for flies bearing the heaviest weight at all three time points. Under these conditions, tarsal contacts were displaced further from the body center. In addition, foreleg and hindleg contacts were shifted anteriorly and posteriorly, respectively. Despite these changes, both AEP and PEP shifted similarly, resulting in an unchanged step length (Figure 4D). For lighter and intermediate weights (0.66× and 1.14×) only small changes were observed in footprint positioning 2 hours after the addition of the weights (Figure 5A). However, 24 hours after addition of the intermediate weight (1.14×), footprint positions shifted to those observed immediately in response to heavier weights (Figure 5B). Thus, adjustments to intermediate weights generally occurred by 24 hours, if not sooner. The spreading of tarsal contacts ensures that the center of mass will remain inside the support area, thus increasing static stability [48]. Footprint clustering also changed in response to increased load (Figure 5D–E). Flies bearing the heaviest weights showed an increase in this parameter, while minimal changes were observed for animals carrying light and intermediate weights, suggesting less stable locomotion under heavier weight-bearing conditions.

Together, these data show that animals change their spatial properties in order to accommodate load increases. The dispersion of tarsal contacts further away from the body center can additionally increase the area of tripod support and thus promote static stability while carrying heavier weights [48].

**Figure 6:**
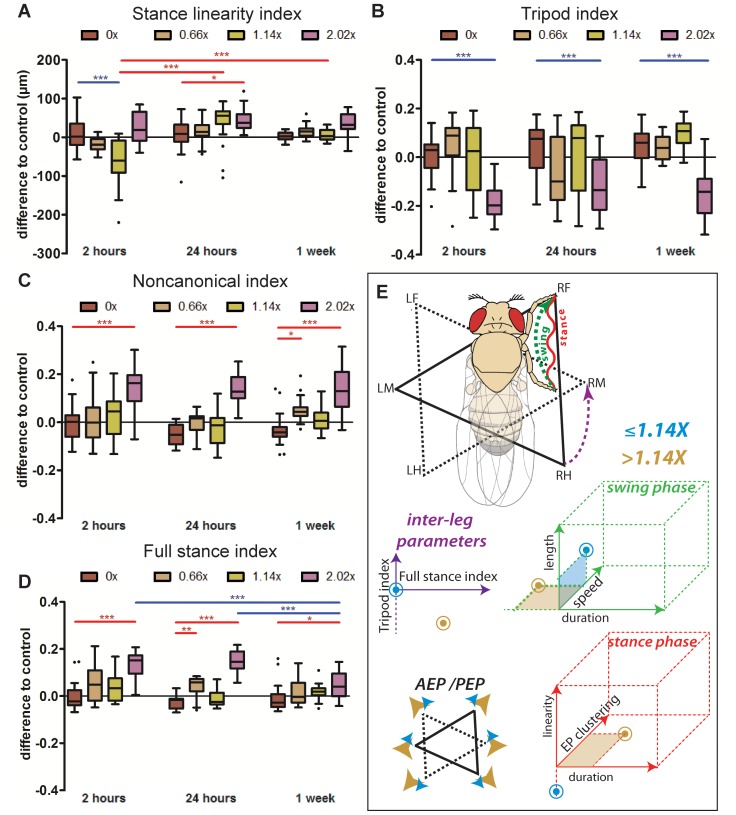
Stance linearity, interleg coordination parameters and summary of kinematic effects under different load conditions. (A–D) Box plots represent the median as the middle line, with the lower and upper edges of the boxes representing the 25% and 75% quartiles, respectively; the whiskers represent the range of the full data set, excluding outliers. Circles indicate outliers. Statistical significance was determined using 2-way-ANOVA with post-hoc t-tests, where ^*^ *p*<0.05; ^**^ *p*<0.01; ^***^ *p*<0.001. Statistically significant increases or decreases are indicated in red and blue, respectively. (A) Stance Linearity Index. Flies bearing intermediate weights show less wobble during stance phases compared to controls at 2 hours, while flies bearing the heaviest loads display more wobble at all time points. (B) Tripod Index. Flies bearing 2.02× weights show significantly reduced tripod index at all three time points. (C) Non-canonical Index is markedly increased for flies bearing 2.02× weights. (D) Full Stance Index is increased for 2.02× bearing flies at all time points. (E) Summary of kinematic affects due to load bearing. For simplicity, only the step cycle of the right foreleg is represented. Dashed green line represents the swing phase from PEP to AEP. Waved red line represents stance traces. Solid triangle represents the tripod conformation formed by RF, LM and RH. Dashed triangle represents the immediately subsequent tripod conformation. Purple dashed line represents the transition between the two tripod conformations. Blue points represent the qualitative effects transiently observed for animals carrying 1.14× or lighter weights, while brown points represent the effects observed for walking animals carrying 2.02× weights.

Stance linearity was also examined under different load conditions. Two hours after load attachment, flies bearing 1.14× and to a lesser extent those bearing 0.66× exhibited a straighter path (smaller stance linearity values; Figure 6A), similar to animals walking in non-upright orientations (Figure 2B). However, this effect was no longer visible after one week. In addition, heavier loads caused a slight increase in stance linearity, further supporting the idea that there exists a tolerance threshold for weight that is between 1.14× and 2.02× the mass of a fly. Footprint alignment values at intermediate speeds (20–34 mm/s) displayed a similar trend: flies carrying intermediate loads exhibited more aligned footsteps compared to flies bearing the heaviest weight (Figure S7C). Additionally, while there was no significant difference observed at 2 hours between flies bearing 2.02× weights and controls, possibly due to the increased variability in the 2 hour controls, a difference was observed at the later time points, when control flies were able to move with increased precision (smaller footprint alignment values) but flies bearing the heaviest weights were not. This trend was also observed in the 1.14× bearing flies at the later time points. These results reinforce a time-dependent kinematic response to weight bearing as well as a different set of responses for flies carrying less than 1.14× compared to flies bearing 2.02× or higher.

### Coordination parameters

To assess whether gaits change upon increased load, we examined the relative frequencies of several step configurations (Figure 6B–D and Figure S7D, E). Quantification of gait patterns showed that animals bearing 1.14× and below did not show any significant alterations in gait conformations. Flies bearing 2.02×, however, displayed significant changes in gait conformations. These flies displayed a strong decrease in the use of the tripod configuration (Figure 6B), and to a lesser extent, tetrapod configurations (Figure S7D). Conversely, we observed a slight increase in the frequency of wave configurations (Figure S7E) and a significant increase in the use of non-canonical configurations (Figure 6C), which includes all possible configurations not covered by tripod, tetrapod and wave patterns. Among these non-canonical configurations, we quantified the fraction of time flies spend with all six legs on the ground while still moving forward, which we refer to as the *full stance index* (Figure 6D). We found that flies bearing 2.02× of body mass spent a significantly increased amount of time in this configuration (Figure 6D). By one week, however, these flies decreased their use of this configuration and returned to control values. Interestingly, this increase in non-canonical gait configurations was not due to an increase in inter-tripod transition time: these values remained relatively constant regardless of the amount of weight being carried and regardless of the amount of time since the weight was added (Figure S7F). Instead, these results suggest that in response to heavier loads, transitions between tripod stances preferentially occur with all six legs on the ground. These results once again highlight a different response to carrying weights 1.14× or below versus 2.02× or above, with minimal effects on gait properties for the former.

### Response to increased load when sensory feedback is compromised

**Figure 7:**
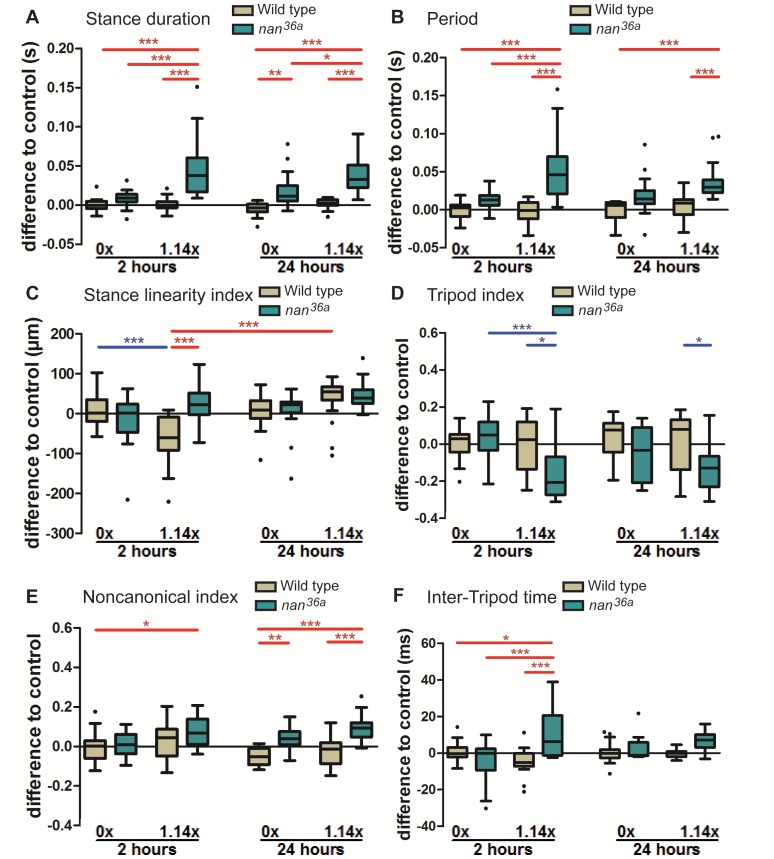
Effects of chordotonal organ deprivation while weight bearing. Box plots represent the median as the middle line, with the lower and upper edges of the boxes representing the 25% and 75% quartiles, respectively; the whiskers represent the range of the full data set, excluding outliers. Circles indicate outliers. Statistical significance was determined using 3-way-ANOVA with post-hoc t-tests, where ^*^ *p*<0.05; ^**^ *p*<0.01; ^***^ *p*<0.001. Statistically significant increases or decreases are indicated in red and blue, respectively. (A) Stance Duration. Only weighted *nan^36a^* flies show increased stance duration, and this effect occurs independently of time. (B) Step period only increases significantly for weighted *nan^36a^* flies in a time-independent manner. (C) Stance linearity index. Animals carrying intermediate weights display a less wobbly stance phase 2 hours after weight bearing, this effect is absent in *nan^36a^* flies. (D) Tripod index. Sensory deprived *nan^36a^* flies display a significant reduction in the use of tripod configurations during weight bearing. (E) Non-canonical index. *nan^36a^* flies weight bearing show an increase in non-canonical configurations in a time-independent way. (F) Inter-Tripod time. Weight bearing *nan^36a^* flies display an increased transition time between tripod configurations. This effect is only visible after 2 hours of weight bearing.

Although campaniform sensilla have been extensively described as the main transducers of load during standing and walking conditions [22]–[25], other proprioceptors could also mediate a response to weight bearing [32]. Here, we tested the role of the femoral chordotonal organ in mediating the kinematic responses to weight bearing. Chordotonal organs are internal stretch receptors composed of multiple functional units termed scolopidia that measure the position and movements of leg joints [1], [52], [53]. We took advantage of a null allele of the *nanchung* (*nan*) gene, which encodes a cation channel subunit responsive to osmotic and mechanical responses and is expressed exclusively in the sensory cilia of chordotonal neurons in the legs and antenna [3], [54]. *nan* mutants disrupt the function of leg chordotonal organs while leaving other leg sensory organs unaffected [39], [54]. We quantified the kinematic effects of carrying loads of 1.14 times body weight in this sensitized genetic background, with measurements done 2 and 24 hours after adding the weight (Figure 7 and S8). Previously we found that loss of chordotonal organ function using the same *nan* allele displayed significant gait and foot placement defects [39]. Although we still detected slower walking speeds for these mutants (Figure S8A), control and *nan* mutant flies exhibited similar walking parameters in the absence of additional load (data not shown), which may be a consequence of the presence of glue on the notums of control flies.

In flies bearing additional weight, *nan* mutants significantly increased stance duration, even 24 hours after the weights were added (Figure 7A). However, no change was observed in swing duration (Figure S8B), resulting in an increased step period (Figure 7B). This result contrasted with that observed in wild type animals, where flies bearing the heaviest weights were nevertheless able to maintain a normal step period due to both an increase in stance duration and a decrease in swing duration (Figure 4E, F). *nan* flies were unable to make this compensatory decrease in swing duration. Moreover, step length and swing speed were no different between control and *nan* mutants bearing intermediate weights (Figure S8C, D). Animals carrying intermediate weights displayed less jitter in their stance phases 2 hours after the loading procedure (Figure 6A). However, this effect was not visible in a *nan* mutant background (Figure 7C), suggesting that this sensory organ can influence motor output allowing stabilization during weight bearing, consistent with the ability of these afferents to synapse directly onto motor neurons [55], [56]. Interleg coordination was also affected in the *nan* mutant leading to a significant reduction of tripod conformations for weight bearing flies at both time points (Figure 7D). While tetrapod configurations seem to be unchanged under these conditions (Figure S8E), there is a compensatory increase in wave and non-canonical configurations (Figure S8F and 7E). While the increase in wave configuration observed in *nan* mutants during weight bearing is only present after 2 hours (Figure S8F), the increase in non-canonical configuration is present both after 2 and 24 hours of weight bearing (Figure 7E). These effects mirror the responses observed when wild type animals carried heavier loads (e.g. compare Figures 6C with 7E). Moreover, *nan* also leads to a significant increase in the transition time from one tripod configuration to the next one (Figure 7F), although this effect is only observed 2 hours after the weights are added. Overall, our results suggest that sensory input, in particular mediated by the chordotonal organ, is used to modulate kinematic parameters in response to increases in gravitational load.

## Discussion

Animals have the remarkable ability to adapt their locomotor behavior to changes in body weight, load and walking orientation. In disorders where motor ability is impaired like Parkinson’s disease, a decreased sensitivity of extensor load reflex mechanisms is observed when compared to healthy individuals. This contributes to the impaired gait in elderly subjects as well as in Parkinsonian patients [57]. Here we determined the kinematic responses upon change in the direction and strength of the gravitational force in freely walking fruit flies. Previous studies have highlighted the ability of the stick insect, cockroach, ant and locust to detect and adapt to changes in walking orientation [42], [43], [46], [58]–[61], focusing on a limited set of kinematic parameters and mostly on ground force reaction and physiological features. These studies have shown an altered recruitment of muscle groups depending on the particular challenges [42], [43], which likely result in distinct kinematic features. In contrast to these model systems, which rely on claws to support inverted walking, fruit flies are equipped with adhesive pads that allow them to easily change their walking orientation under natural and laboratory conditions [12], [13]. These distinct strategies might also contribute to differences in locomotor behavior.

Our data reveal that fruit flies walking under non-upright conditions display differences in their gait properties, suggesting that these responses are the consequence of orientation-specific neuronal programs suited to specific walking conditions. Interestingly, these responses do not affect the duration of the power stroke (the stance phase; Figure 1F) but rather target the stride (step length; Figure 1D) and the duration of the swing phase (Figure 1E). Moreover, in inverted walking animals this shift in step length and swing duration lead to a significant increase in swing speed (Figure 1C), which, as pointed out by Larsen et al., may reflect the animal’s choice to maximize leg contact with the substrate in order to minimize instability [43]. In accordance with a more controlled approach for locomotion under non-upright conditions, we observed that under these situations, stance phases show less wobble (lower stance linearity values) compared to flies walking in an upright position (Figure 2B). This effect could be the consequence of a more controlled stance phase in which larger or more motor units become recruited, leading to muscular co-contraction of antagonistic muscles. As has been observed in the locust, this promotes joint stiffness in order to sustain gait stability and a constant distance from the body to the substrate [42]. However, recruitment of additional motor units would be expected to result in a more consistent placement and removal of tarsal contacts during the step cycle, but no difference was observed in footprint clustering either for AEP or PEP between upright and non-upright conditions in our experiments (Figure 2C, D).

We were also able to observe changes in interleg coordination in response to changes in walking orientation (Figure 3). Modulation of interleg coordination is used to sustain speed, stability and energy efficiency [38], [39], [47]–[49]. Most noticeably, in our experiments inverted-walking animals displayed a shift from anchoring on three legs (the tripod gait) to five legs (typical of wave gait). Similarly, in the stick insect, the canonical tripod and tetrapod configurations were present in different fractions compared to “irregular” gaits whenever the animals moved across inclined substrates [61]. Nevertheless, the tripod gait was still preferred in all conditions, particularly at higher speeds, but with one important difference: the time taken to transition from one tripod configuration to the next was significantly increased (Figure 3E, F). We suggest that changes in interleg configurations allow more time for gait transitions, thus minimizing the chances of contact loss with the substrate. These changes in step strategies in non-upright conditions are done at the expense of speed and possibly energy consumption, partially explaining the reduced and more restricted distribution of walking speeds (Figure 1B).

We also examined the kinematic effects to long-term increases of in gravitational load (Figures 4–7). Although the weight tolerance displayed by *Drosophila* is much lower than that displayed by rhinoceros beetles, which can carry more than 30 times their body mass [15], it is still greater than humans. For example, an average soldier of 80 kg can carry up to 55 kg in load (∼0.7x body mass), causing extreme strain and affecting gait and performance [62]. Our experiments tried to address three questions. First, what are the kinematic responses to an increase in gravitational load? Second, how do these responses change over time? And third, are kinematic responses proportional to load increase or is the response non-linear, reaching a limit of tolerance? Our experiments clearly show that fruit flies can tolerate a considerable range of loads with minimal kinematic consequences. Moreover, some kinematic adjustments change over time suggesting that flies have the capacity to adapt to carrying additional weight.

Importantly, we find that for a variety of kinematic parameters, flies bearing 1.14× or below responded differently from flies bearing 2.02×. For example, when carrying 1.14× their body weight, flies showed a decrease in speed with gradual recovery over time, strongly suggesting that flies can physically deal with such a load (Figure 4B). However, when challenged with heavier loads such a recovery was not observed. In fact, when carrying 2.02× their weight, most kinematic parameters differed significantly from the control group at all time points. Values obtained for average speed (Figure 4B), footprint clustering (Figure 5D, E) and stance linearity index (Figure 6A) demonstrate that somewhere between 1.14 and 2.02 their body weight, there exists a threshold above which flies are much less able to walk in a coordinated manner. Beyond this weight threshold, physical constraints prevent the fly’s neuro-muscular system to function properly. Similar threshold-dependent kinematic and physiological effects have been seen in human weight lifting, where increases in spinal load are observed only when boxes above 20 kilograms are lifted [63].

Our results also highlight some of the ways in which flies promote stability while carrying weights (major kinematic shifts are summarized in Figure 6E). These include an increase in swing speed and a higher duty factor (Figures 4C and S7A) which, interestingly, were also observed in flies walking upside-down, suggesting that these may be commonly used solutions to deal with a variety of challenging situations. Moreover, weight bearing also promoted tarsal contacts to be positioned further away from the body (Figure 5A–C) thus leading to an increased area of support and static stability [48]. Animals carrying extra weight also shifted their interleg coordination parameters such that during tripod transition periods all legs were often in contact with the substrate.

In response to carrying 2.02× their body weight, some kinematic parameters, including average speed (Figure 4B); swing speed (Figure 4C) and footprint positioning (Figure 5A–C), remained different from those of control animals, even after one week. This trend could be the result of physical fatigue. Interestingly, it was not observed in animals carrying the lighter and intermediate loads (0.66× and 1.14×), which could sustain their walking speed even after one week of load bearing (Figure 4B). However, we observed a decrease in the full stance index after one week for flies bearing the heaviest weight. The fact that these flies showed a reduction in full stance phase suggests that they were eventually able to at least partially adapt to the heavy load. In sum, our observations suggest that these long-term effects are the result of an adaptive process that is less robust for flies carrying the heaviest of weights. Motor neurons and CPGs exhibit neuronal plasticity in order to cope with new challenges. Such an adaptive process may be analogous to spinal cord injury models, where recovery has been suggested to depend on locomotor training and CPG plasticity [64], [65].

Our data also suggest a role for the leg chordotonal organ as a sensor for gravitational load (Figure 7 and S8). In flies with this proprioceptor impaired, we observed an increased sensitivity to lighter loads and an inability to cope with these loads. In addition, *nanchung* mutant flies did not survive long-term exposure to intermediate weights, further supporting the importance of proprioception in weight bearing (data not shown). The leg chordotonal organs are highly sensitive to angle variations in the femur-tibia and tibia-tarsus joints caused by increases in gravitational load which feedback to preserve posture, thus indirectly reporting gravitation load variation. Similarly to the campaniform sensilla, the femoral chordotonal organ can also target both motor neurons and spiking and non-spiking interneurons [55], [56], indicating this proprioceptor can promote reflex responses upon weight bearing. Work in the stick insect has also shown that the campaniform sensilla can modify the reflex pathway mediated by the femoral chordotonal organ, suggesting that these two organs act cooperatively [66]. Thus, these data suggest the chordotonal organ can assist the campaniform sensilla to resolve weight detection. It is also plausible that the femoral chordotonal organ directly acts as a sensor of body load. Consistent with this idea, the dendrites of the femoral chordotonal organ are attached to a connective tissue ligament that inserts into a cuticular apodeme in the proximal tibia [55], [67], detecting forces in the femoral-tibia joint and making this organ mechanically competent to detect strains caused by load variations [55], [56], [66]. Other insect proprioceptors have been suggested to mediate responses to weight bearing such as multipolar muscle tension receptors present in the cockroach, stick insect and locust [67]–[69]. However, these structures have not been identified in *Drosophila*.

In summary, our results underscore the ability of multi-jointed organisms to respond kinematically to changes in walking conditions. Such plasticity is dependent on sensory input, which can directly or indirectly target pattern generators. Moreover, adaptability may not be immediate (reflex-based) but rather a time dependent process possibly involving several locomotor centers or even genetic networks. Future studies should address what neuronal units and genes are responsible for such adaptability. Finally, it is worth noting that the strategies and mechanisms displayed by walking organisms can be implemented in artificial neuronal networks, which are able to autonomously control walking machines in a stable, adaptable and energy efficient fashion [70]–[72].

## Materials and Methods

### Fly strains

Wild type (Oregon R) and nan^36a^ flies were reared on standard cornmeal at 25°C. All flies used in this study were non-virgin females between 1 and 7 days old. All manipulations were carried out under cold anesthesia. For the experiments described in Figures 1–3, at least 12 independent trials were conducted for each experimental condition, with each trial including 3–5 flies. On average, ∼3 videos per trial were recorded and analyzed, for a total of at least 23 videos. For the more technically challenging experiments described in Figures 4–7, ≥5 flies were examined for each experimental condition, for a total of at least 20 videos. In one condition (0.66× weight at 1 week), 20 videos were generated from three independent animals.

### fTIR imaging and FlyWalker software

For the walking orientation experiments the fTIR setup was re-arranged so that the fly could be visualized walking inverted on a ceiling or vertically (descending or ascending; Figure 1A). For the load experiments flies were imaged 2 hours, 24 hours, and 1 week after load attachment. Videos were quantified for kinematics using the FlyWalker software package. See [39] for details. In addition to the previously published kinematic parameters, four additional parameters were quantified:

#### Wave index

Fraction of frames in a video that display leg combinations defined by the wave (or metachronal) gait [50].

#### Full stance index

Fraction of frames in a video in which the body is moving forward while all six legs are in a stance phase.

#### Tripod duration

Average duration of a single tripod stance. Only tripod stances that had at least three consecutive frames with the same leg combination were considered.

#### Inter-tripod time

Average time to transition from one tripod stance to the next tripod stance. Only tripod stances lasting three or more consecutive frames were considered. In addition, only videos that contained 20% or more of tripod and 40% or less tetrapod configurations were considered.

### Attachment of body load

1/32″ spherical ball bearings were used as body loads. Aluminum and tungsten carbide ball bearings were obtained from McMaster-Carr Supply Co. (New Jersey, USA). Brass ball bearings were obtained from Salem Specialty Ball Co. (Connecticut, USA). Aluminum, tungsten carbide and brass ball bearings were determined to be about 0.66, 1.14 and 2.02 times the weight of an individual female fly, respectively. Each fly was considered to have 1.14±0.05 mg (n = 39). Flies were cold anesthetized throughout the load attachment procedure. Ball bearings were attached to the fly notum under a dissection microscope using a UV-curable glue from Loctite. Weight loading took about 30 seconds per fly. Loaded flies were kept on standard cornmeal at 25°C with vials turned horizontally so that they were free to access the food while minimizing the possibility of falling into the food. They were allowed 2 hours to recover after load attachment.

### Statistical analysis

Each data point comes from a single video. Since many of the measured gait parameters vary with speed (Swing speed, Step length, Stance duration, Stance linearity, Footprint clustering and gait indexes), we analyzed the data for these parameters by determining the best-fit regression model for the control experiment and then determining the residual values for each experimental group in relation to this regression model. Data was then expressed as the difference to the residual-normalized line. Boxplots represent the median as the middle line, with the lower and upper edges of the boxes representing the 25% and 75% quartiles, respectively; the whiskers represent the range of the full data set, excluding outliers. Outliers are defined as any value that is 1.5 times the interquartile range below of above the 25% and 75% quartiles, respectively. Statistical differences between experimental groups in Figures 1–3 were determined using Kruskal-Wallis analysis of variance (ANOVA) followed by Dunn’s *post hoc* test (for non-normal distributions) or one-way-ANOVA followed by Tukey’s *post hoc* test (for normal distributions). For figures 4–7, S7 and S8, tests for significance were done using a 2-way or 3-wayANOVA followed by Tukey’s post-hoc test (GraphPad Prism).

## Supporting Information

Figure S1 **Gait parameters by walking orientation.** (A–E) Each column corresponds to a walking orientation compared to upright controls. Graphical fits are also represented. (A) Swing speed. (B) Step length. (C) Swing duration. (D) Stance duration. (E) Duty factor.
(TIF)

Figure S2 **Stance linearity and footprint alignment by walking orientation.** Each column corresponds to a walking orientation compared to upright controls. Graphical fits are also represented. (A) Stance linearity. (B) Footprint alignment.
(TIF)

Figure S3 **Footprint clustering by walking orientation.** Each column corresponds to a walking orientation compared to upright controls. Graphical fits are also represented. (A–C) Anterior Extreme Position (AEP) clustering. (D–F) Posterior Extreme Position (PEP) clustering. (A, D) Forelegs. (B, E) Midlegs. (C, F) Hindlegs.
(TIF)

Figure S4 **Footprint positions relative to the body center for the different walking orientations.** AEP and PEP values for each leg are represented on the left and right sections of the plot, respectively. Values are normalized for body size. Line size denotes standard deviations, while intersection indicates mean value. (A) Inverted. (B) Ascending. (C) Descending. Statistical significance was determined using 2-way-ANOVA with Tukey’s post-hoc tests and post-hoc t-tests, where *p<0.05; **p<0.01; ***p<0.001.
(TIF)

Figure S5 **Interleg coordination indexes by walking orientation.** Each column corresponds to a walking orientation compared to upright controls. (A) Tripod index. (B) Tetrapod index. (C) Wave index. (D) Non-canonical index.
(TIF)

Figure S6 **Inter Tripod time and tripod duration by walking orientation.** Each column corresponds to a walking orientation compared to upright controls. Graphical fits are also represented. (A) Inter Tripod time (B) Tripod duration.
(TIF)

Figure S7 (A) Duty factor. (B) Period. (C) Footprint Alignment. (D) Tetrapod index. (E) Wave Index. (F) Inter-tripod time. Box plots represent the median as the middle line, with the lower and upper edges of the boxes representing the 25% and 75% quartiles, respectively; the whiskers represent the range of the full data set, excluding outliers. Circles indicate outliers. Statistical significance was determined using 2-way-ANOVA with post-hoc t-tests, where *p<0.05; **p<0.01; ***p<0.001. Statistically significant increases or decreases are indicated in red and blue, respectively.
(TIF)

Figure S8 (A) Average Speed. (B) Swing Duration. (C) Step Length. (D) Swing Speed. (E) Tetrapod Index. (F) Wave Index. Box plots represent the median as the middle line, with the lower and upper edges of the boxes representing the 25% and 75% quartiles, respectively; the whiskers represent the range of the full data set, excluding outliers. Circles indicate outliers. Statistical significance was determined using 3-way-ANOVA with post-hoc t-tests, where *p<0.05; **p<0.01; ***p<0.001. Statistically significant increases or decreases are indicated in red and blue, respectively.
(TIF)

Table S1 **Parameter definitions and units used in the text.**
(DOCX)

Video S1 **Representative video of a fly carrying a metal ball bearing.**
(AVI)

## References

[1] Kernan MJ (2007) Mechanotransduction and auditory transduction in Drosophila. Pflugers Arch 454: 703–720. 17436012 10.1007/s00424-007-0263-x

[2] Büschges A, DiCaprio RA (2008) 6.17 - Somatosensation in Invertebrates. In: Volume Editors: Allan IB, Akimichi K, Gordon MS, Gerald W, Thomas DA et al.., editors. The Senses: A Comprehensive Reference. New York: Academic Press. 355–362.

[3] Sun Y, Liu L, Ben-Shahar Y, Jacobs JS, Eberl DF, et al (2009) TRPA channels distinguish gravity sensing from hearing in Johnston's organ. Proc Natl Acad Sci U S A 106: 13606–13611. 19666538 10.1073/pnas.0906377106 PMC2717111

[4] Kamikouchi A, Inagaki HK, Effertz T, Hendrich O, Fiala A, et al (2009) The neural basis of Drosophila gravity-sensing and hearing. Nature 458: 165–171. 19279630 10.1038/nature07810

[5] Kaplan WD, Trout WE (1974) Genetic manipulation of an abnormal jump response in Drosophila. Genetics 77: 721–739. 4371648 10.1093/genetics/77.4.721 PMC1213163

[6] Tanouye MA, Wyman RJ (1980) Motor outputs of giant nerve fiber in Drosophila. J Neurophysiol 44: 405–421. 6774064 10.1152/jn.1980.44.2.405

[7] Koto M, Tanouye MA, Ferrus A, Thomas JB, Wyman RJ (1981) The morphology of the cervical giant fiber neuron of Drosophila. Brain Res 221: 213–217. 6793208 10.1016/0006-8993(81)90772-1

[8] Allen MJ, Godenschwege TA, Tanouye MA, Phelan P (2006) Making an escape: development and function of the Drosophila giant fibre system. Semin Cell Dev Biol 17: 31–41. Epub 2005 Dec 2027. 10.1016/j.semcdb.2005.11.011 16378740

[9] Pick S, Strauss R (2005) Goal-driven behavioral adaptations in gap-climbing Drosophila. Curr Biol 15: 1473–1478. 16111941 10.1016/j.cub.2005.07.022

[10] Triphan T, Poeck B, Neuser K, Strauss R (2010) Visual targeting of motor actions in climbing Drosophila. Curr Biol 20: 663–668. 20346674 10.1016/j.cub.2010.02.055

[11] Bender JA, Frye MA (2009) Invertebrate solutions for sensing gravity. Curr Biol 19: R186–190 doi:110.1016/j.cub.2008.1012.1024 19278626 10.1016/j.cub.2008.12.024

[12] Gorb SN, Beutel RG (2001) Evolution of locomotory attachment pads of hexapods. Naturwissenschaften 88: 530–534. 11824227 10.1007/s00114-001-0274-y

[13] Niederegger S, Gorb S (2003) Tarsal movements in flies during leg attachment and detachment on a smooth substrate. J Insect Physiol 49: 611–620. 12804721 10.1016/s0022-1910(03)00048-9

[14] Moll K, Roces F, Federle W (2013) How load-carrying ants avoid falling over: mechanical stability during foraging in Atta vollenweideri grass-cutting ants. PLoS One 8: e52816 doi:52810.51371/journal.pone.0052816. Epub 0052013 Jan 0052812 23300994 10.1371/journal.pone.0052816 PMC3534694

[15] Kram R (1996) Inexpensive load carrying by rhinoceros beetles. J Exp Biol 199: 609–612. 9318326 10.1242/jeb.199.3.609

[16] Full RJ, Blickhan R, Ting LH (1991) Leg design in hexapedal runners. J Exp Biol 158: 369–390. 1919412 10.1242/jeb.158.1.369

[17] Chang YH, Huang HW, Hamerski CM, Kram R (2000) The independent effects of gravity and inertia on running mechanics. J Exp Biol 203: 229–238. 10607533 10.1242/jeb.203.2.229

[18] Jamon M (2014) The development of vestibular system and related functions in mammals: impact of gravity. Front Integr Neurosci 8: 11. 24570658 10.3389/fnint.2014.00011 PMC3916785

[19] Matsuo E, Kamikouchi A (2013) Neuronal encoding of sound, gravity, and wind in the fruit fly. J Comp Physiol A Neuroethol Sens Neural Behav Physiol 199: 253–262. 23494584 10.1007/s00359-013-0806-x

[20] Windhorst U (2007) Muscle proprioceptive feedback and spinal networks. Brain Res Bull 73: 155–202. 17562384 10.1016/j.brainresbull.2007.03.010

[21] Burrows M (1996) The neurobiology of an insect brain: Oxford University Press.

[22] Pringle JWS (1938) Proprioception In Insects: II. The Action Of The Campaniform Sensilla On The Legs. Journal of Experimental Biology 15: 114–131.

[23] Noah JA, Quimby L, Frazier SF, Zill SN (2001) Force detection in cockroach walking reconsidered: discharges of proximal tibial campaniform sensilla when body load is altered. J Comp Physiol A 187: 769–784. 11800034 10.1007/s00359-001-0247-9

[24] Noah JA, Quimby L, Frazier SF, Zill SN (2004) Sensing the effect of body load in legs: responses of tibial campaniform sensilla to forces applied to the thorax in freely standing cockroaches. J Comp Physiol A Neuroethol Sens Neural Behav Physiol 190: 201–215. Epub 2004 Jan 2016. 10.1007/s00359-003-0487-y 14727134

[25] Keller BR, Duke ER, Amer AS, Zill SN (2007) Tuning posture to body load: decreases in load produce discrete sensory signals in the legs of freely standing cockroaches. J Comp Physiol A Neuroethol Sens Neural Behav Physiol 193: 881–891. Epub 2007 Jun 2001. 10.1007/s00359-007-0241-y 17541783

[26] Ridgel AL, Frazier SF, Zill SN (2001) Dynamic responses of tibial campaniform sensilla studied by substrate displacement in freely moving cockroaches. J Comp Physiol A 187: 405–420. 11529484 10.1007/s003590100213

[27] Burrows M (1985) The processing of mechanosensory information by spiking local interneurons in the locust. J Neurophysiol 54: 463–478. 4045534 10.1152/jn.1985.54.3.463

[28] Burrows M, Pfluger HJ (1988) Positive feedback loops from proprioceptors involved in leg movements of the locust. Journal of Comparative Physiology A.

[29] Zill SN, Moran DT, Varela FG (1981) The Exoskeleton and Insect Proprioception: II. Reflex Effects of Tibial Campaniform Sensilla in the American Cockroach, Periplaneta Americana. Journal of Experimental Biology 94: 43–55.

[30] Akay T, Bassler U, Gerharz P, Buschges A (2001) The role of sensory signals from the insect coxa-trochanteral joint in controlling motor activity of the femur-tibia joint. J Neurophysiol 85: 594–604. 11160496 10.1152/jn.2001.85.2.594

[31] Akay T, Ludwar B, Goritz ML, Schmitz J, Buschges A (2007) Segment specificity of load signal processing depends on walking direction in the stick insect leg muscle control system. J Neurosci 27: 3285–3294. 17376989 10.1523/JNEUROSCI.5202-06.2007 PMC6672458

[32] Zill S, Schmitz J, Buschges A (2004) Load sensing and control of posture and locomotion. Arthropod Struct Dev 33: 273–286. 18089039 10.1016/j.asd.2004.05.005

[33] Full RJ, Tu MS (1991) Mechanics of a rapid running insect: two-, four- and six-legged locomotion. J Exp Biol 156: 215–231. 2051129 10.1242/jeb.156.1.215

[34] Jindrich DL, Full RJ (1999) Many-legged maneuverability: dynamics of turning in hexapods. J Exp Biol 202: 1603–1623. 10333507 10.1242/jeb.202.12.1603

[35] Quimby LA, Amer AS, Zill SN (2006) Common motor mechanisms support body load in serially homologous legs of cockroaches in posture and walking. J Comp Physiol A Neuroethol Sens Neural Behav Physiol 192: 247–266. Epub 2005 Dec 2016. 10.1007/s00359-005-0062-9 16362305

[36] Venken KJT, Simpson JH, Bellen HJ (2011) Genetic Manipulation of Genes and Cells in the Nervous System of the Fruit Fly. Neuron 72: 202–230. 22017985 10.1016/j.neuron.2011.09.021 PMC3232021

[37] Strauss R, Heisenberg M (1990) Coordination of legs during straight walking and turning in Drosophila melanogaster. J Comp Physiol [A] 167: 403–412. 10.1007/BF00192575 2121965

[38] Wosnitza A, Bockemuhl T, Dubbert M, Scholz H, Buschges A (2013) Inter-leg coordination in the control of walking speed in Drosophila. J Exp Biol 216: 480–491 doi:410.1242/jeb.078139. Epub 072012 Oct 078134 23038731 10.1242/jeb.078139

[39] Mendes CS, Bartos I, Akay T, Marka S, Mann RS (2013) Quantification of gait parameters in freely walking wild type and sensory deprived Drosophila melanogaster. elife 2: e00231. 23326642 10.7554/eLife.00231 PMC3545443

[40] Branson K, Robie AA, Bender J, Perona P, Dickinson MH (2009) High-throughput ethomics in large groups of Drosophila. Nat Methods 6: 451–457. 19412169 10.1038/nmeth.1328 PMC2734963

[41] Kabra M, Robie AA, Rivera-Alba M, Branson S, Branson K (2013) JAABA: interactive machine learning for automatic annotation of animal behavior. Nat Methods 10: 64–67. 23202433 10.1038/nmeth.2281

[42] Duch C, Pfl&Uuml, Ger H (1995) Motor patterns for horizontal and upside down walking and vertical climbing in the locust. J Exp Biol 198: 1963–1976. 9319873 10.1242/jeb.198.9.1963

[43] Larsen GS, Frazier SF, Fish SE, Zill SN (1995) Effects of load inversion in cockroach walking. J Comp Physiol A 176: 229–238. 7884685 10.1007/BF00239925

[44] Cruse H, Dean J, Suilmann M (1984) The contributions of diverse sense organs to the control of leg movement by a walking insect. Journal of Comparative Physiology A: 695–705.

[45] Song SM, Choi BS (1989) A study on continuous follow-the-leader (FTL) gaits: an effective walking algorithm over rough terrain. Math Biosci 97: 199–233. 2520211 10.1016/0025-5564(89)90005-9

[46] Cruse H (1976) The function of the legs in the free walking stick insect, Carausius morosus. 235–262.

[47] Hoyt DF, Taylor R (1981) Gait and the energetics of locomotion in horses. Nature 292: 239–240.

[48] Ting LH, Blickhan R, Full RJ (1994) Dynamic and static stability in hexapedal runners. J Exp Biol 197: 251–269. 7852905 10.1242/jeb.197.1.251

[49] Bender JA, Simpson EM, Tietz BR, Daltorio KA, Quinn RD, et al (2011) Kinematic and behavioral evidence for a distinction between trotting and ambling gaits in the cockroach Blaberus discoidalis. J Exp Biol 214: 2057–2064. 21613522 10.1242/jeb.056481 PMC3102013

[50] Graham D, BerridgeMJ, Treherne JE, Wigglesworth VB (1985) Pattern and Control of Walking in Insects. Advances in Insect Physiology: Academic Press. 31–140.

[51] Full RJ, Koditschek DE (1999) Templates and anchors: neuromechanical hypotheses of legged locomotion on land. J Exp Biol 202: 3325–3332. 10562515 10.1242/jeb.202.23.3325

[52] Hofmann T, Koch UT, Bässler U (1985) Physiology of the Femoral Chordotonal Organ in the Stick Insect, Cuniculina Impigra. Journal of Experimental Biology 114: 207–223.

[53] Büschges A (1994) The physiology of sensory cells in the ventral scoloparium of the stick insect femoral chordotonal organ. The Journal of Experimental Biology 189: 285–292. 9317814 10.1242/jeb.189.1.285

[54] Kim J, Chung YD, Park DY, Choi S, Shin DW, et al (2003) A TRPV family ion channel required for hearing in Drosophila. Nature 424: 81–84. 12819662 10.1038/nature01733

[55] Burrows M (1987) Parallel processing of proprioceptive signals by spiking local interneurons and motor neurons in the locust. J Neurosci 7: 1064–1080. 3572474 10.1523/JNEUROSCI.07-04-01064.1987 PMC6568994

[56] Hess D, Buschges A (1997) Sensorimotor pathways involved in interjoint reflex action of an insect leg. J Neurobiol 33: 891–913. 9407012

[57] Dietz V, Colombo G (1998) Influence of body load on the gait pattern in Parkinson's disease. Mov Disord 13: 255–261. 9539338 10.1002/mds.870130210

[58] Full RJ, Tullis A (1990) Energetics of ascent: insects on inclines. J Exp Biol 149: 307–317. 2324672 10.1242/jeb.149.1.307

[59] Lipp A, Wolf H, Lehmann FO (2005) Walking on inclines: energetics of locomotion in the ant Camponotus. J Exp Biol 208: 707–719. 15695763 10.1242/jeb.01434

[60] Seidl T, Wehner R (2008) Walking on inclines: how do desert ants monitor slope and step length. Front Zool 5: 8. 18518946 10.1186/1742-9994-5-8 PMC2430559

[61] Grabowska M, Godlewska E, Schmidt J, Daun-Gruhn S (2012) Quadrupedal gaits in hexapod animals – inter-leg coordination in free-walking adult stick insects. The Journal of Experimental Biology 215: 4255–4266. 22972892 10.1242/jeb.073643

[62] Knapik JJ, Reynolds KL, Harman E (2004) Soldier load carriage: historical, physiological, biomechanical, and medical aspects. Mil Med 169: 45–56. 14964502 10.7205/milmed.169.1.45

[63] Davis KG, Marras WS (2000) The effects of motion on trunk biomechanics. Clin Biomech (Bristol, Avon) 15: 703–717. 10.1016/s0268-0033(00)00035-8 11050352

[64] Rossignol S, Brustein E, Bouyer L, Barthelemy D, Langlet C, et al (2004) Adaptive changes of locomotion after central and peripheral lesions. Can J Physiol Pharmacol 82: 617–627. 15523519 10.1139/y04-068

[65] Muir GD (1999) Locomotor plasticity after spinal injury in the chick. J Neurotrauma 16: 705–711. 10511243 10.1089/neu.1999.16.705

[66] Akay T, Buschges A (2006) Load signals assist the generation of movement-dependent reflex reversal in the femur-tibia joint of stick insects. J Neurophysiol 96: 3532–3537. 16956989 10.1152/jn.00625.2006

[67] Matheson T, Field L (1995) An elaborate tension receptor system highlights sensory complexity in the hind leg of the locust. J Exp Biol 198: 1673–1689. 9319581 10.1242/jeb.198.8.1673

[68] Guthrie DM (1967) Multipolar stretch receptors and the insect leg reflex. Journal of Insect Physiology 13: 1637–1644.

[69] Bässler U (1977) Sense organs in the femur of the stick insect and their relevance to the control of position of the femur-tibia-joint. Journal of comparative physiology 121: 99–113.

[70] Cruse H, Kindermann T, Schumm M, Dean J, Schmitz J (1998) Walknet-a biologically inspired network to control six-legged walking. Neural Netw 11: 1435–1447. 12662760 10.1016/s0893-6080(98)00067-7

[71] Durr V, Schmitz J, Cruse H (2004) Behaviour-based modelling of hexapod locomotion: linking biology and technical application. Arthropod Struct Dev 33: 237–250. 18089037 10.1016/j.asd.2004.05.004

[72] Cruse H, Durr V, Schmitz J (2007) Insect walking is based on a decentralized architecture revealing a simple and robust controller. Philos Transact A Math Phys Eng Sci 365: 221–250. 10.1098/rsta.2006.1913 17148058

[73] Simon JC, Dickinson MH (2010) A new chamber for studying the behavior of Drosophila. PLoS ONE 5: e8793. 20111707 10.1371/journal.pone.0008793 PMC2811731

