# Supplementary figures and images for: Kinematic Responses to Changes in Walking Orientation and Gravitational Load in Drosophila melanogaster
Source: PLoS One. 2014 Oct 28;9(10):e109204. doi: 10.1371/journal.pone.0109204 (PMC4211655; doi:10.1371/journal.pone.0109204)

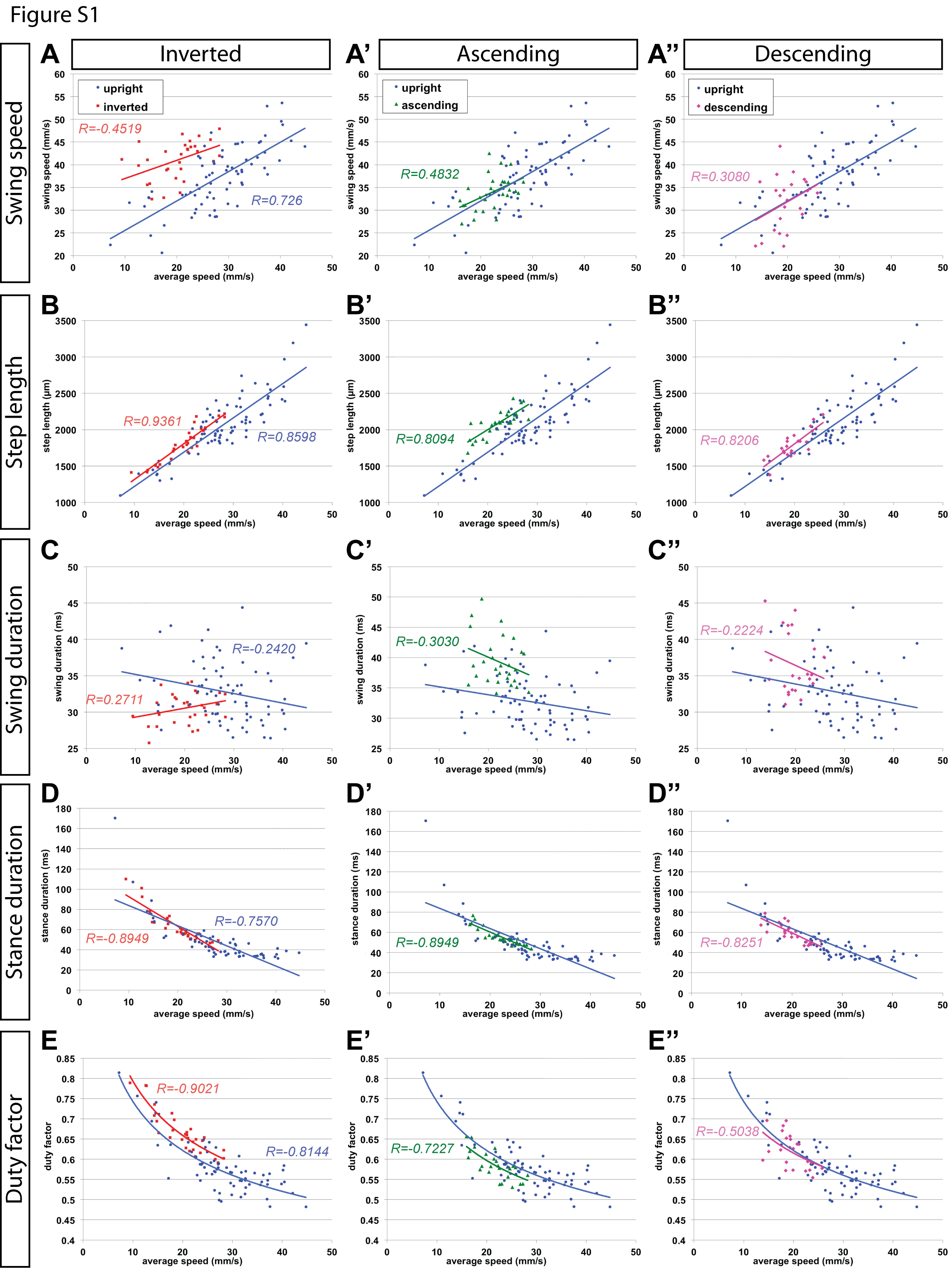

Supplement: Figure S1 — Gait parameters by walking orientation. (A–E) Each column corresponds to a walking orientation compared to upright controls. Graphical fits are also represented. (A) Swing speed. (B) Step length. (C) Swing duration. (D) Stance duration. (E) Duty factor. (TIF) [file pone.0109204.s001.tif]

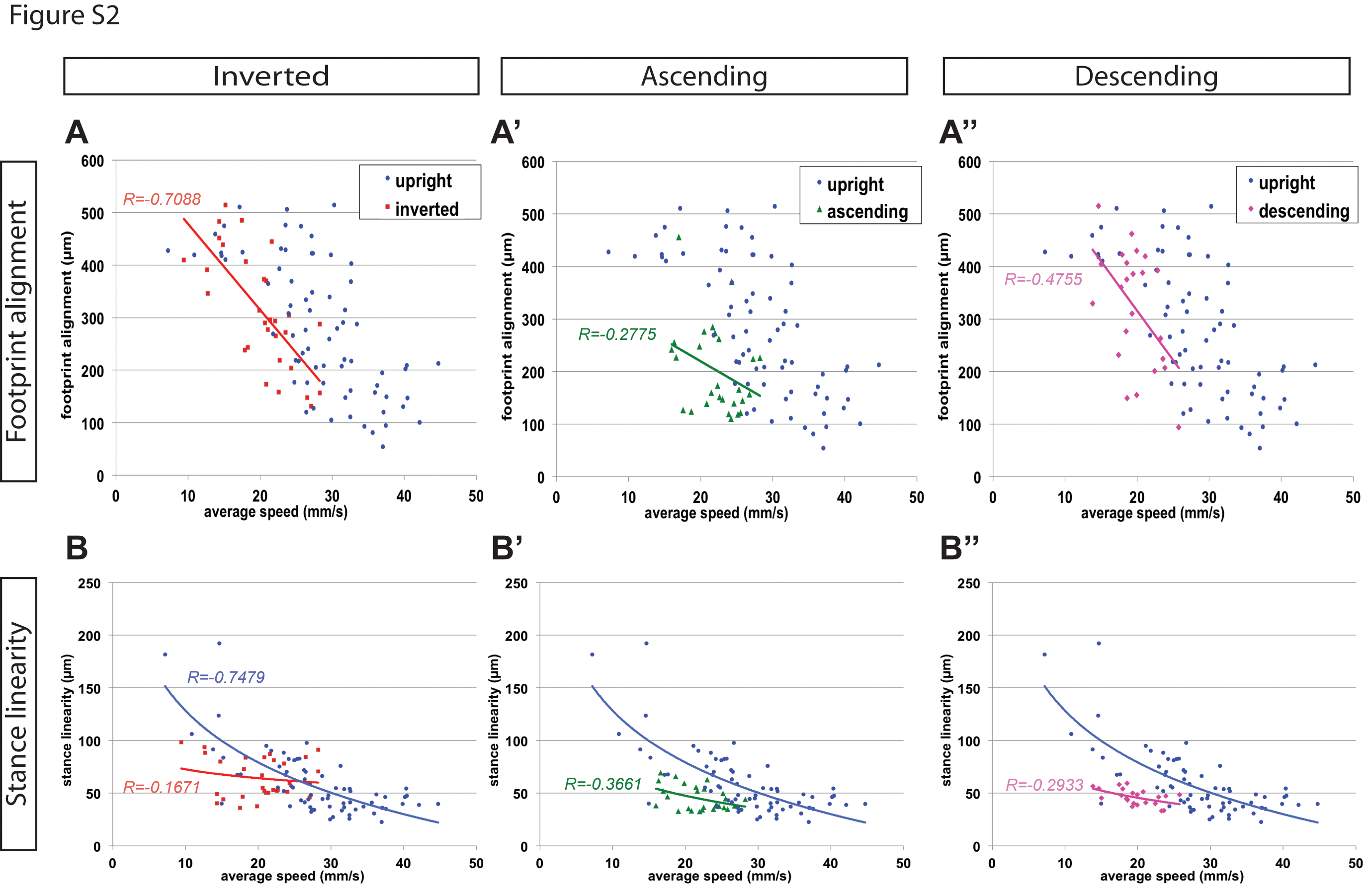

Supplement: Figure S2 — Stance linearity and footprint alignment by walking orientation. Each column corresponds to a walking orientation compared to upright controls. Graphical fits are also represented. (A) Stance linearity. (B) Footprint alignment. (TIF) [file pone.0109204.s002.tif]

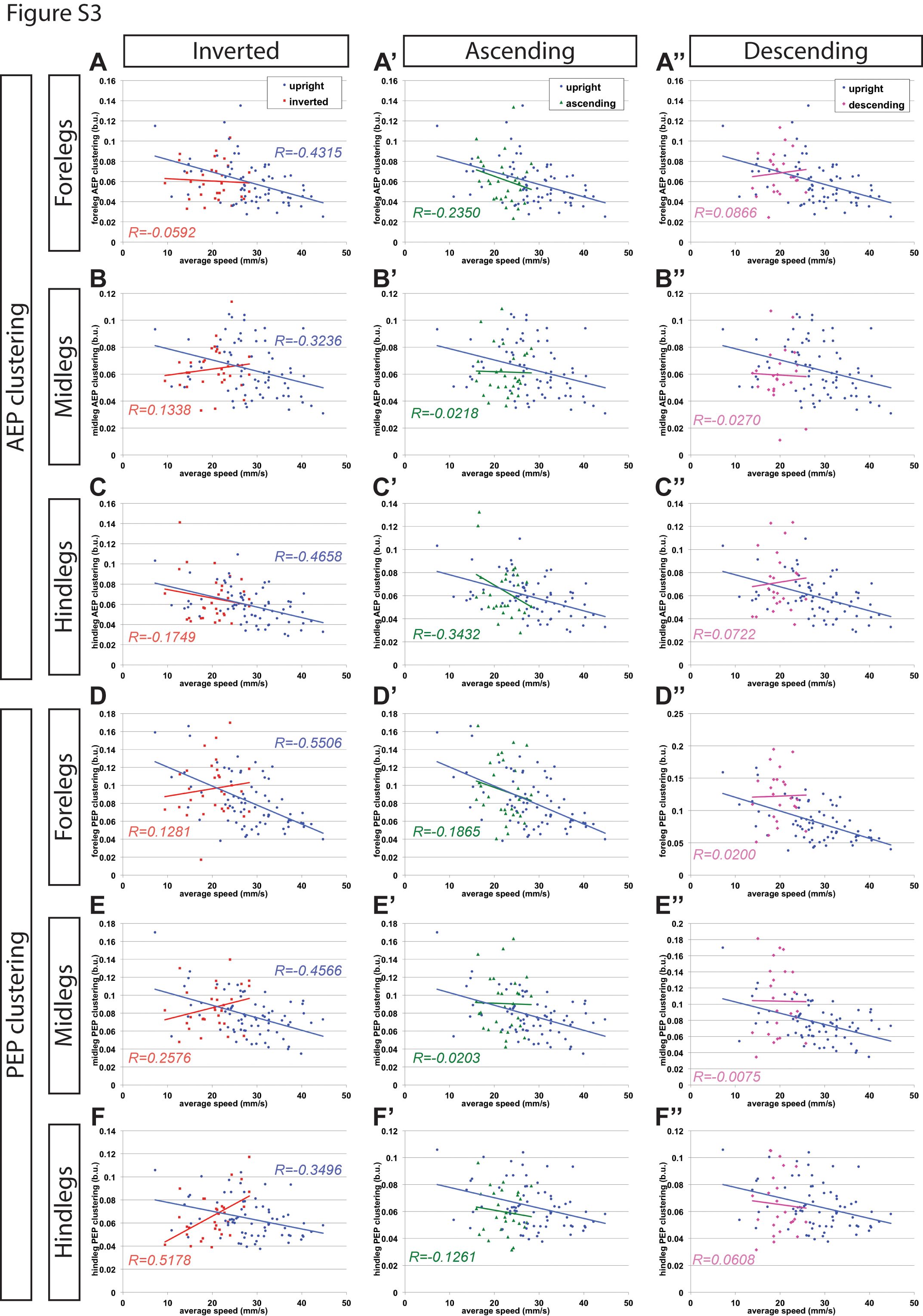

Supplement: Figure S3 — Footprint clustering by walking orientation. Each column corresponds to a walking orientation compared to upright controls. Graphical fits are also represented. (A–C) Anterior Extreme Position (AEP) clustering. (D–F) Posterior Extreme Position (PEP) clustering. (A, D) Forelegs. (B, E) Midlegs. (C, F) Hindlegs. (TIF) [file pone.0109204.s003.tif]

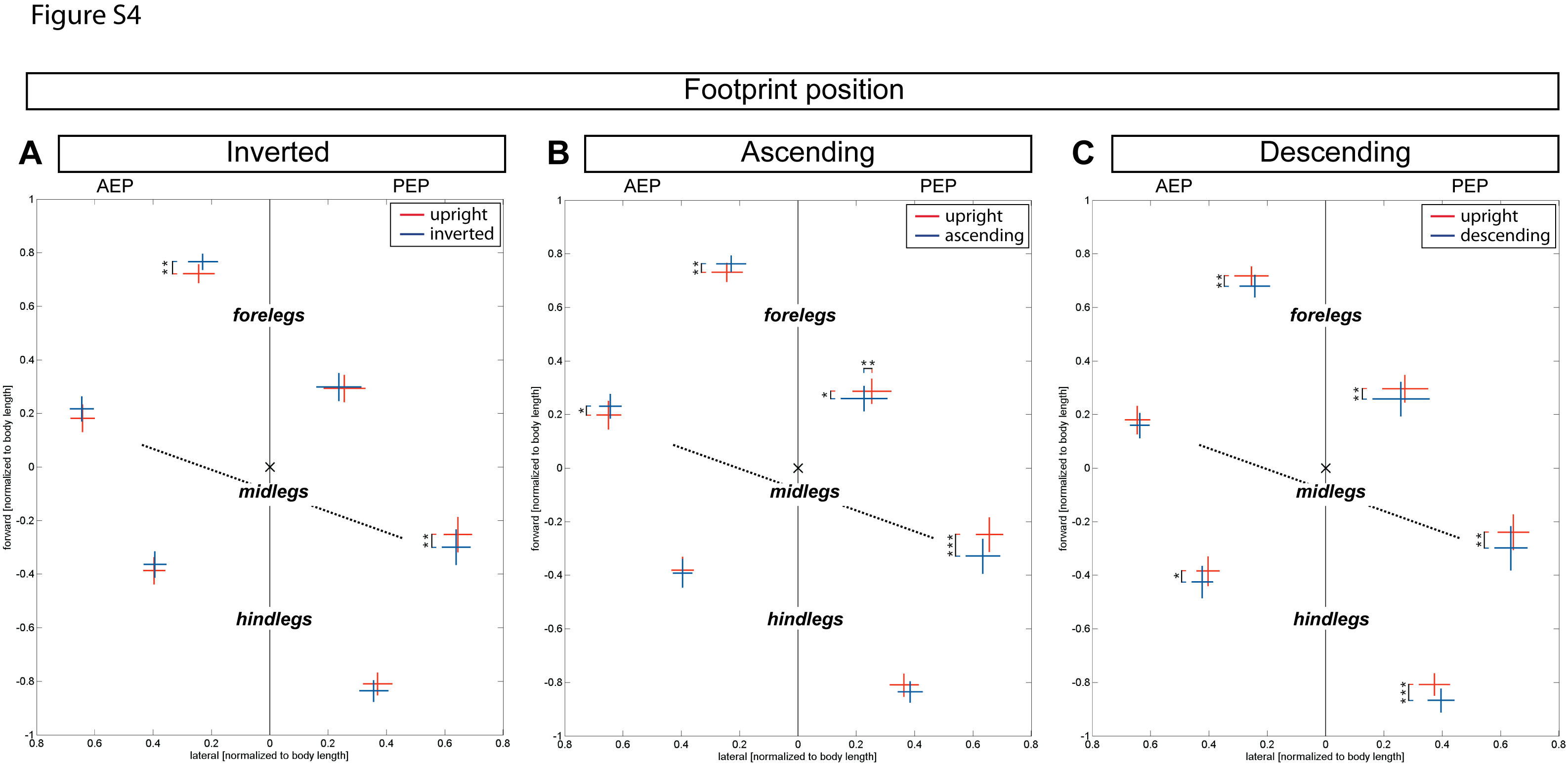

Supplement: Figure S4 — Footprint positions relative to the body center for the different walking orientations. AEP and PEP values for each leg are represented on the left and right sections of the plot, respectively. Values are normalized for body size. Line size denotes standard deviations, while intersection indicates mean value. (A) Inverted. (B) Ascending. (C) Descending. Statistical significance was determined using 2-way-ANOVA with Tukey’s post-hoc tests and post-hoc t-tests, where *p<0.05; **p<0.01; ***p<0.001. (TIF) [file pone.0109204.s004.tif]

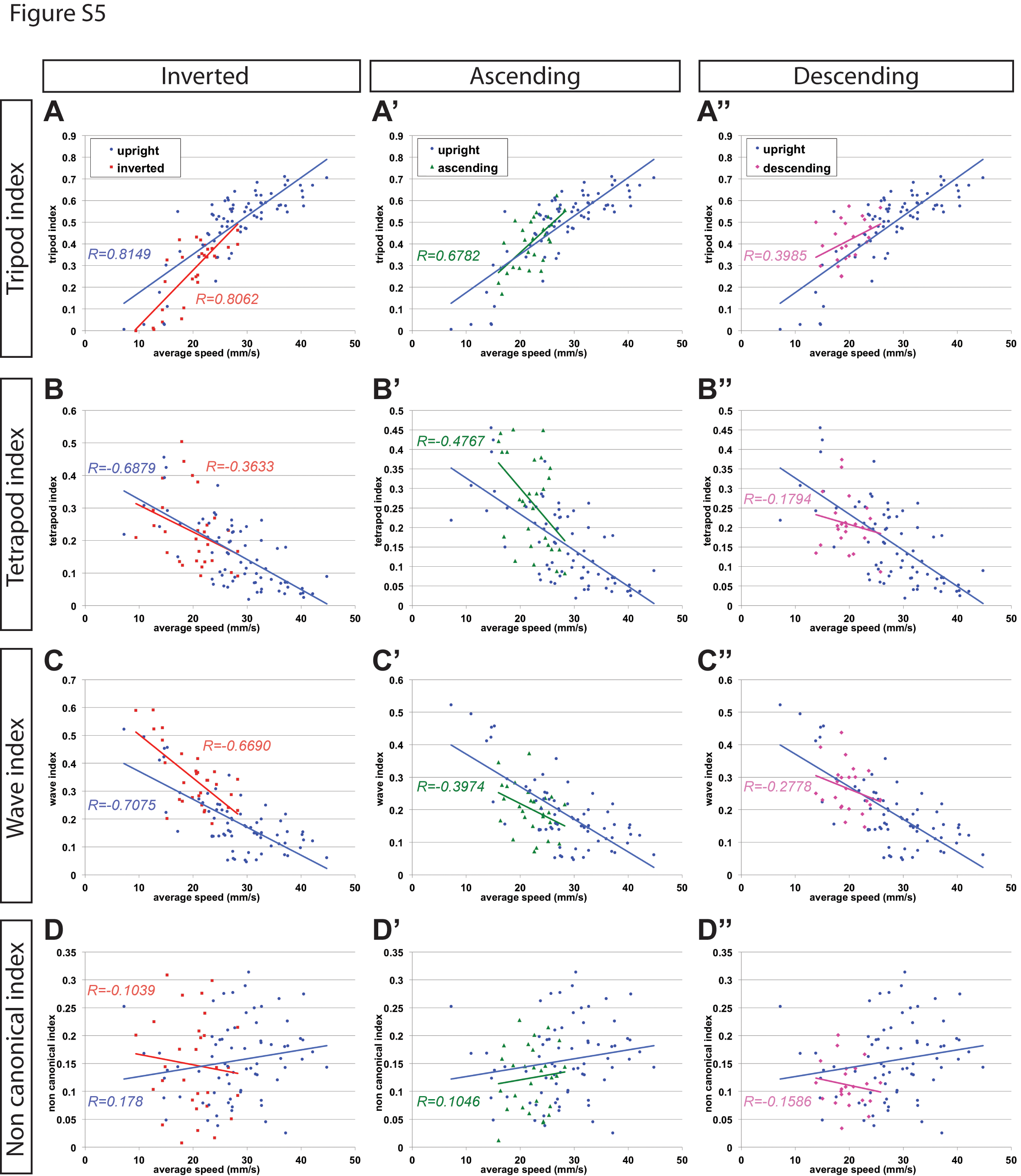

Supplement: Figure S5 — Interleg coordination indexes by walking orientation. Each column corresponds to a walking orientation compared to upright controls. (A) Tripod index. (B) Tetrapod index. (C) Wave index. (D) Non-canonical index. (TIF) [file pone.0109204.s005.tif]

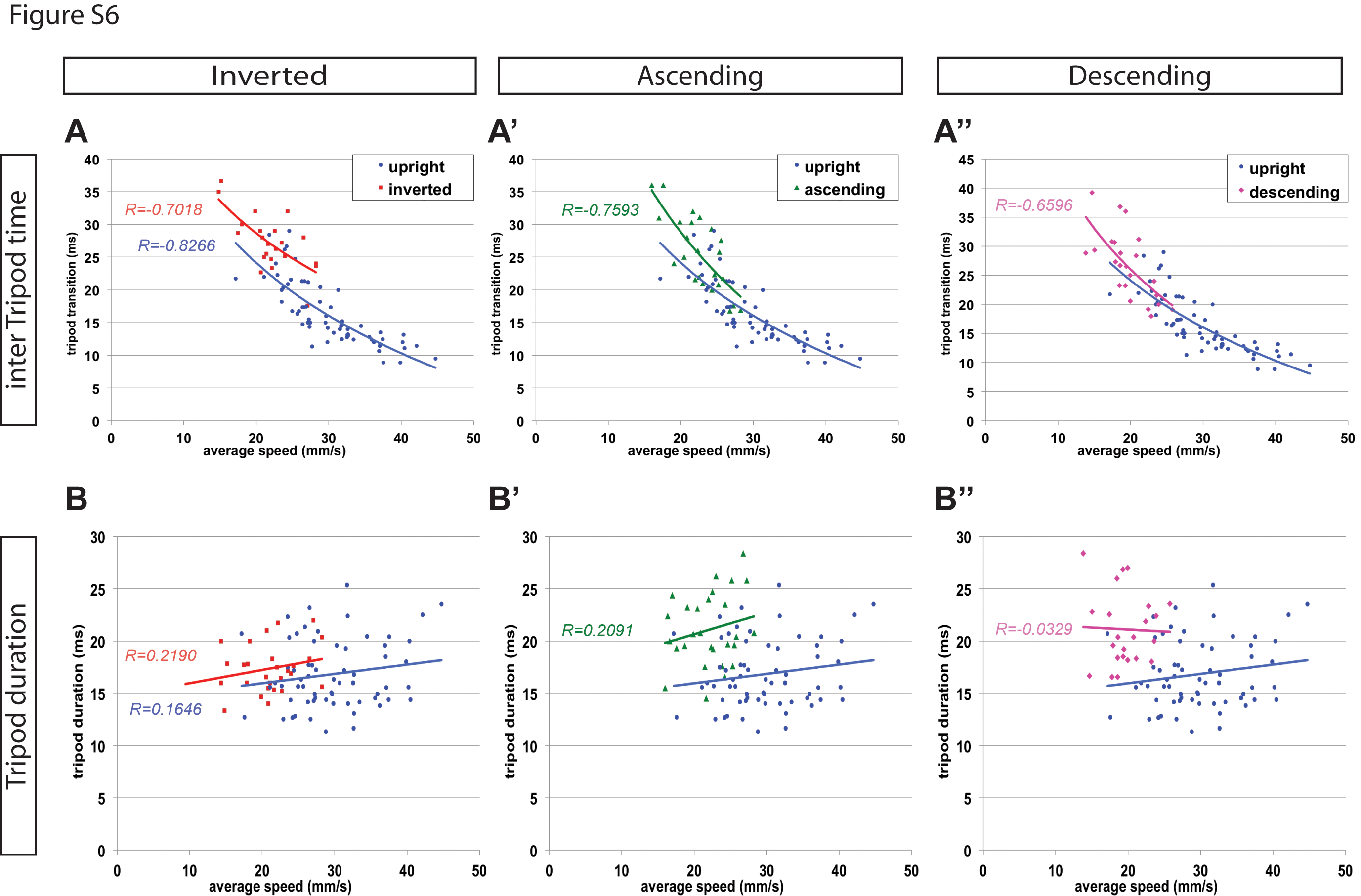

Supplement: Figure S6 — Inter Tripod time and tripod duration by walking orientation. Each column corresponds to a walking orientation compared to upright controls. Graphical fits are also represented. (A) Inter Tripod time (B) Tripod duration. (TIF) [file pone.0109204.s006.tif]

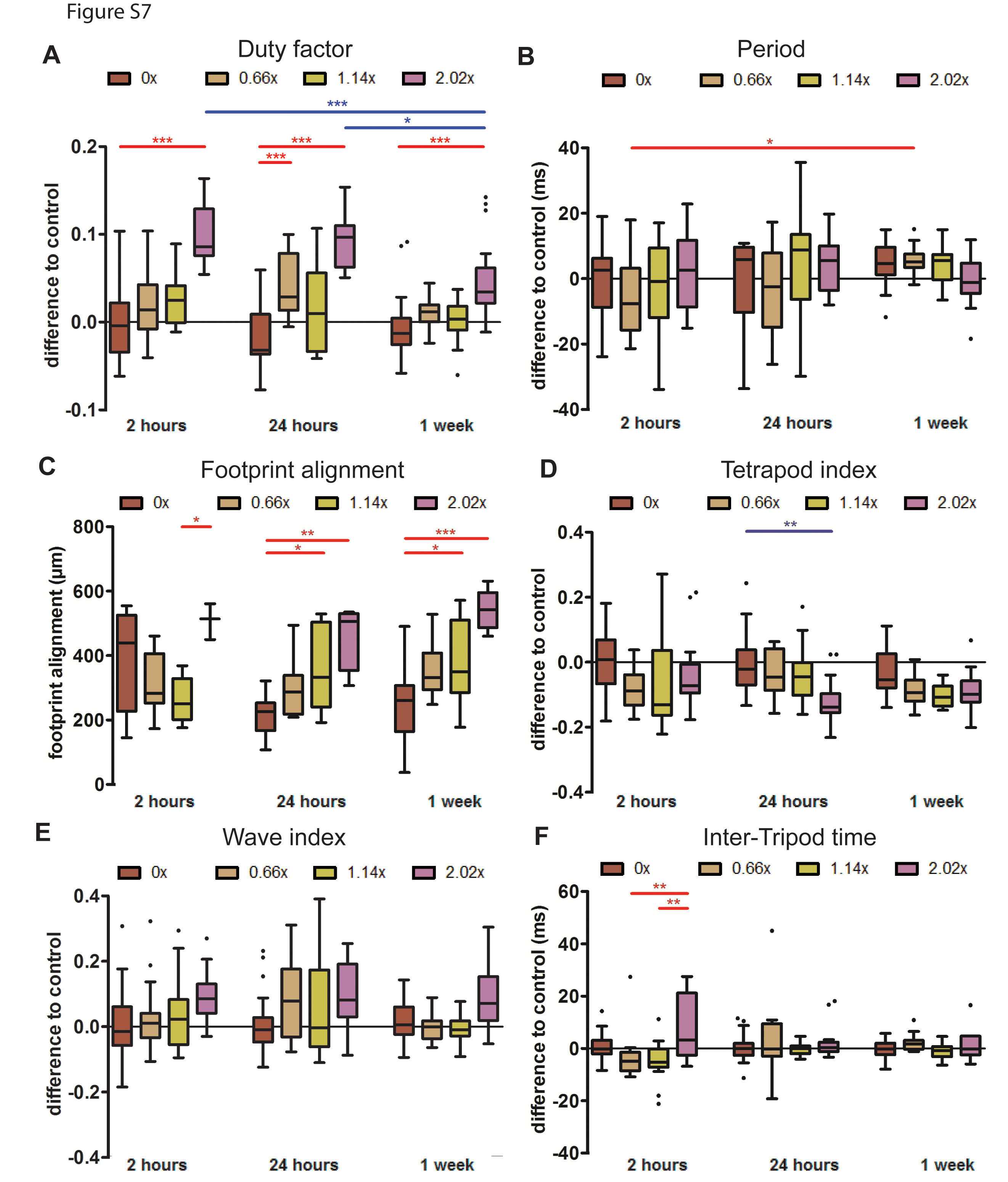

Supplement: Figure S7 — (A) Duty factor. (B) Period. (C) Footprint Alignment. (D) Tetrapod index. (E) Wave Index. (F) Inter-tripod time. Box plots represent the median as the middle line, with the lower and upper edges of the boxes representing the 25% and 75% quartiles, respectively; the whiskers represent the range of the full data set, excluding outliers. Circles indicate outliers. Statistical significance was determined using 2-way-ANOVA with post-hoc t-tests, where *p<0.05; **p<0.01; ***p<0.001. Statistically significant increases or decreases are indicated in red and blue, respectively. (TIF) [file pone.0109204.s007.tif]

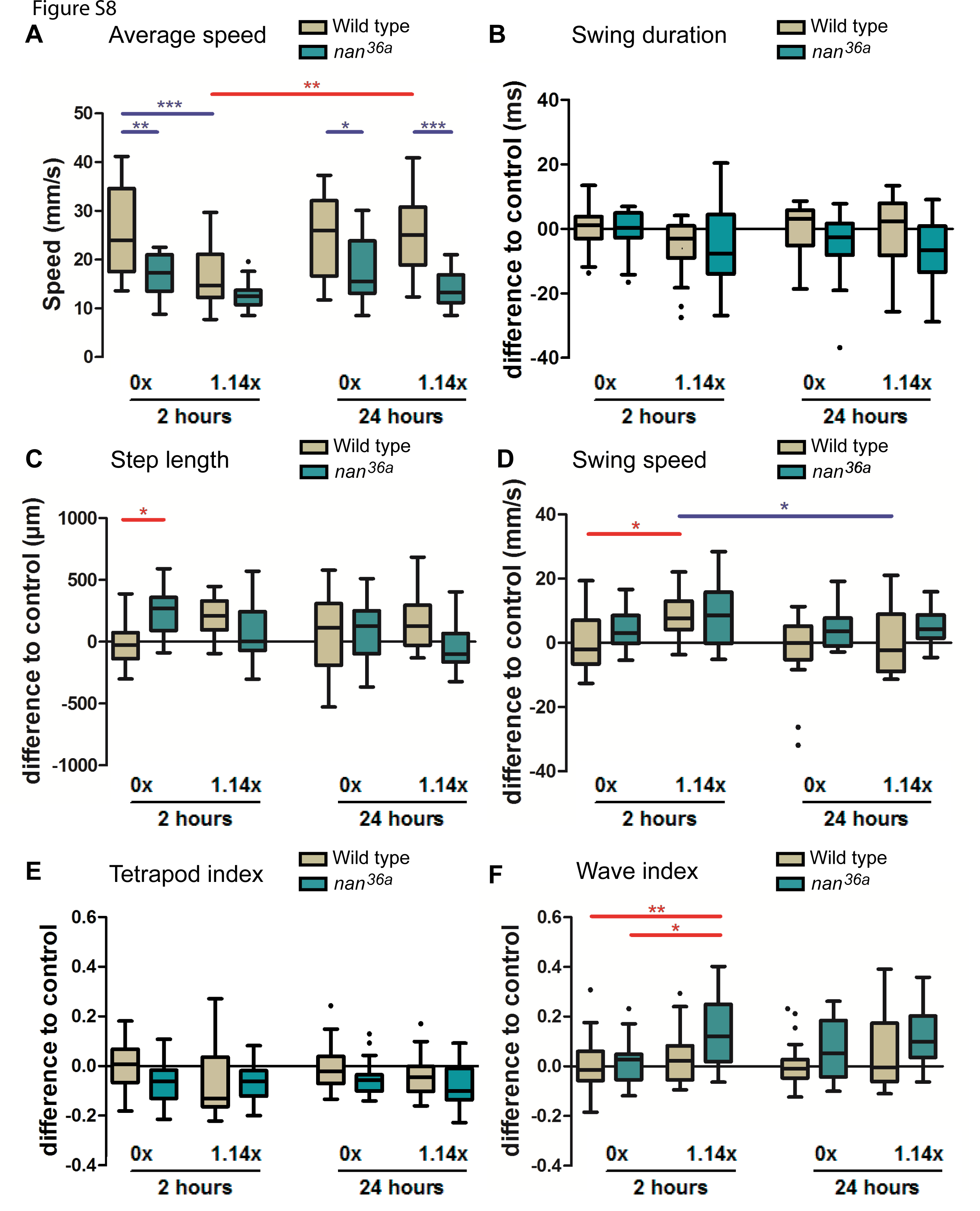

Supplement: Figure S8 — (A) Average Speed. (B) Swing Duration. (C) Step Length. (D) Swing Speed. (E) Tetrapod Index. (F) Wave Index. Box plots represent the median as the middle line, with the lower and upper edges of the boxes representing the 25% and 75% quartiles, respectively; the whiskers represent the range of the full data set, excluding outliers. Circles indicate outliers. Statistical significance was determined using 3-way-ANOVA with post-hoc t-tests, where *p<0.05; **p<0.01; ***p<0.001. Statistically significant increases or decreases are indicated in red and blue, respectively. (TIF) [file pone.0109204.s008.tif]
