# Supplementary material for: Kinematic Responses to Changes in Walking Orientation and Gravitational Load in Drosophila melanogaster
Source: PLoS One. 2014 Oct 28;9(10):e109204. doi: 10.1371/journal.pone.0109204 (PMC4211655; doi:10.1371/journal.pone.0109204)
Supplement: Table S1 — Parameter definitions and units used in the text. (DOCX) [file pone.0109204.s009.docx]

**Table S1.** Parameter definitions.

Terms and definitions used:

***Average speed*** (mm/s): Speed of the fly averaged across an entire video, without any stops.

***Period*** (ms): Time taken to complete one leg cycle consisting of one swing and one stance phase.

***Step*** ***length*** (μm): Distance between two successive footprints of the same leg. No normalization in relation of the direction of propagation.

***Swing duration*** (ms): Time between swing onset and offset for two successive footprints of the same leg.

***Stance duration*** (ms): Time between stance onset and offset for the same footprint.

***Swing*** ***speed*** (mm/s): Step length divided by swing duration, using the body center as reference.

***Duty factor*** (unitless): Stance duration divided by period. Measures the fraction of the step cycle dedicated to the stance phase.

***Stance Linearity Index*** (μm): Average difference between the stance traces generated by each leg during stance phase and a 5-point smoothed line.

***Anterior Extreme Position* (*AEP*)** (body units): Position where the leg first contacts the glass after touchdown at the end of swing phase (or protraction), relative to body center.

***Posterior Extreme Position* (*PEP*)** (body units): Position at the end of the stance phase, just before the tarsi enter swing phase, relative to body center.

***Footprint clustering*** (body units): Standard deviation from the average position for all *AEP*s or *PEP*s in a single video.

***Tripod index*** (unitless): Fraction of frames in a video that display leg combinations defined by the tripod gait.

***Tetrapod index*** (unitless): Fraction of frames in a video that display leg combinations defined by the tetrapod gait.

***Wave index*** (unitless)*:* Fraction of frames in a video that display leg combinations defined by the wave (or metachronal) gait.

***Full stance index*** (unitless)*:* Fraction of frames in a video in which the body is moving forward while all six legs are in a stance phase.

***Tripod duration*** (ms): Average duration of each tripod stance in a video.

***Inter-Tripod time*** (ms): Average time taken to transition from one tripod stance to the next tripod stance.

***Footprint alignment*** (μm): Standard deviation from the average point of adjacent ipsilateral footprints projected onto the displacement axis. Each set of ipsilateral footprints includes one foreleg, one midleg and one hindleg.

Abbreviations: s, second; ms, millisecond; μm, micrometer; mm, millimeter; body unit, distance relative to the length of the body, with the center of mass set at 0.
